# Shaking hands is a homeodomain transcription factor that controls axon outgrowth of central complex neurons in the insect model *Tribolium*

**DOI:** 10.1242/dev.199368

**Published:** 2021-10-13

**Authors:** Natalia Carolina Garcia-Perez, Gregor Bucher, Marita Buescher

**Affiliations:** Johann Friedrich Blumenbach Institute of Zoology, GZMB, Department of Evolutionary Developmental Genetics, University of Goettingen, Justus-von-Liebig Weg 11, 37077 Goettingen, Germany

**Keywords:** *Tribolium* brain, Central complex development, Homeodomain transcription factor

## Abstract

Gene regulatory mechanisms that specify subtype identity of central complex (CX) neurons are the subject of intense investigation. The CX is a compartment within the brain common to all insect species and functions as a ‘command center’ that directs motor actions. It is made up of several thousand neurons, with more than 60 morphologically distinct identities. Accordingly, transcriptional programs must effect the specification of at least as many neuronal subtypes. We demonstrate a role for the transcription factor Shaking hands (Skh) in the specification of embryonic CX neurons in *Tribolium*. The developmental dynamics of *skh* expression are characteristic of terminal selectors of subtype identity. In the embryonic brain, *skh* expression is restricted to a subset of neurons, many of which survive to adulthood and contribute to the mature CX. *skh* expression is maintained throughout the lifetime in at least some CX neurons. *skh* knockdown results in axon outgrowth defects, thus preventing the formation of an embryonic CX primordium. The previously unstudied *Drosophila skh* shows a similar embryonic expression pattern, suggesting that subtype specification of CX neurons may be conserved.

## Abstract (precis)

**Summary:** A detailed examination of the developmental expression of the homeodomain transcription factor Shaking hands in *Tribolium* reveals a role in the formation of the central complex primordium.

## INTRODUCTION

The insect brain contains a large number of neurons with distinct identities. Cell identity is manifest in specific structural and functional features, which together define a neuronal subtype. Subtype specification is already completed in the newborn neuron and, during differentiation, it effects axon pathfinding, thus facilitating the formation of specific neural connections. Neuronal subtypes express distinct sets of differentiation genes, which together bring about all the characteristic features of the cell. Transcription factors that regulate the expression of differentiation genes are the endpoint of hierarchical gene regulatory cascades that act earlier during development (Hobert, 2008, 2011; Allan and Thor, 2015; Hobert and Kratsios, 2019). The early regulatory cascades that govern neuronal subtype specification have been intensively investigated in the insect model *Drosophila melanogaster* (reviewed by Skeath and Thor, 2003; Lin and Lee, 2012; Crews, 2019). All cells of the *Drosophila* brain derive from embryonically born stem cells, called neuroblasts (NBs). Each NB gives rise to a stereotyped and invariant lineage of neurons and glia. Each NB has a unique identity that manifests in the expression of a unique combination of transcription factors (Urbach and Technau, 2003). NB identity is determined by overlapping spatial information in the procephalic neuroectoderm. Additional neuronal diversity is generated by a temporal cascade: each NB expresses distinct transcription factors in an invariant temporal series. Temporal factors are inherited by the NB progeny and establish neuronal cell fates characteristic for a given temporal window (Kohwi and Doe, 2013; Lin and Lee, 2012; Rossi et al., 2017; Doe, 2017). The expression of temporal transcription factors can be transient, making them unlikely regulators of differentiation genes that need to be expressed throughout the life of a neuron. In the *Drosophila* ventral nerve cord (VNC), spatial and temporal factors converge to activate the expression of transcription factors that function as terminal selectors of neuronal subtype identity: these factors regulate the lifelong expression of effector genes, which together bring about all features of the differentiated cell type (Allan and Thor, 2015; Hobert and Kratsios, 2019).

**Fig. 1.**
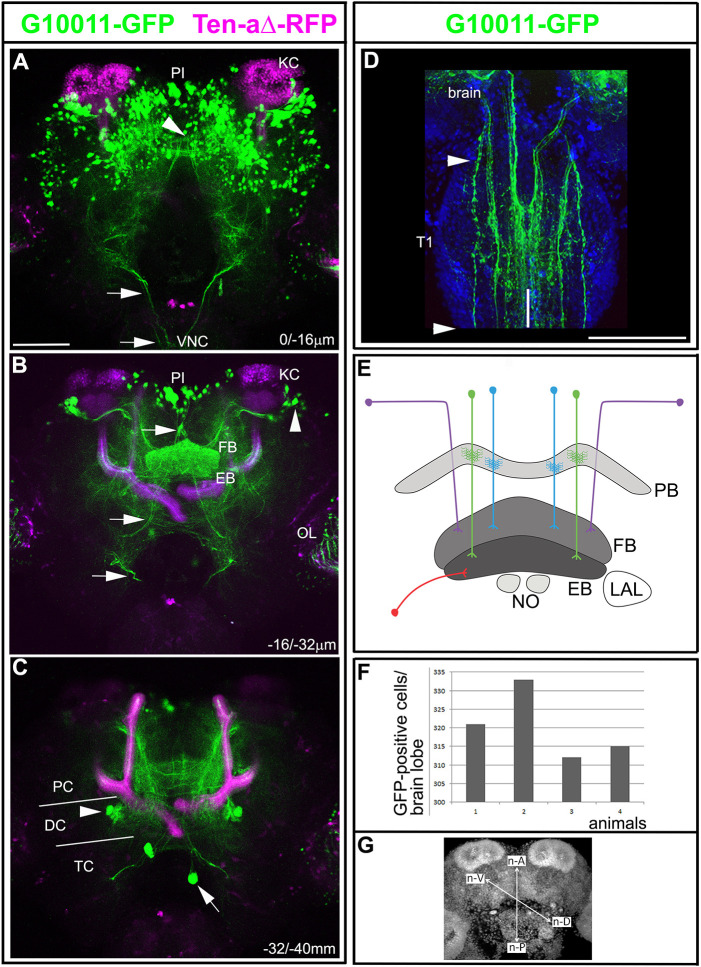
**The enhancer trap line G10011-GFP labels CX neuropils in the adult *Tribolium* brain.** Brain of an animal with the genotype G10011-GFP;Ten-aΔ-RFP (GFP autofluorescence is green and RFP autofluorescence is magenta). In the central brain, Ten-aΔ-RFP expression is restricted to the MBs (magenta). Serial confocal sections were combined and visualized as maximum intensity projections to display distinct anatomical features. Scan direction is from the n-D to the n-V surface of the brain (coordinates of the neuraxes are shown in G). Depth along the *z*-axis is given in µm. (A) GFP-positive cell bodies in the posterior brain. GFP expression is absent from the KCs of the MBs. Arrowhead indicates the PB, which is only partially visible (for the PB, refer to Fig. 2A). Arrows indicate descending axon projections, which extend longitudinal connectives into the VNC. (B) The FB and the EB are heavily labeled by GFP. Clusters of laterally located cells send their axon trajectories toward the upper layer of the FB body (arrowhead; compare with Fig. 2L). Small clusters of large cells are located in the PI. Based on location and axon projections, they may be neurosecretory cells. They project axon tracts towards the VNC (arrows). GFP fluorescence is also seen in the OLs. (C) The arrowhead marks a single cell cluster of four-to-six GFP-positive cells in the anterior brain. Individual large cells within the TC project anteriorly towards the PI (arrow). (D) First thoracic segment (T1) of the VNC: multiple axon projections that originate in the brain form longitudinal connectives in the VNC (arrowheads mark the limits of the first thoracic segment T1). There is an absence of GFP-positive somata in the VNC. Vertical white line marks the ventral midline. (E) Schematic of CX small-field and large-field neurons. Two types of small-field neuron, pb-fb-eb (green) and pb-fb (blue), are shown. Two types of large-field neuron, a ring neuron (red) and AOTU neurons (purple), are shown. (F) Quantification of the number of GFP-positive cells in the adult brain. The number of G10011-GFP-positive cells was 320 per brain lobe (mean of four animals, 1-4). (G) Coordinates according to the neuraxes. Scale bars: 100 μm (A-D). DC, deutocerebrum; n-A, n-anterior; n-D, n-dorsal; n-P, n-posterior; n-V, n-ventral; OL, optic lobe; PC, protocerebrum; TC, tritocerebrum.

The specification of the subtype identity of neurons the trajectories of which comprise the central complex (CX) is a topic of current interest (Boyan and Reichert, 2011; Sullivan et al., 2019; Hartenstein et al., 2020). The CX is a compartment in the center of the brain common to all insect species. It functions as a multi-modal information-processing center that commands locomotor behaviors (Strauss and Heisenberg, 1993; Pfeiffer and Homberg, 2014; Heinze, 2017; Franconville et al., 2018). Anatomically, the adult CX is an ensemble of interconnected paired and unpaired neuropils (Hanesch et al., 1989; Strausfeld, 1999). Core components are the protocerebral bridge (PB), the fan-shaped body (FB), the ellipsoid body (EB) and the noduli (NO) (Fig. 1E). The PB is located at the dorsoposterior cell body-neuropil interface, wedged between the two calyces of the mushroom bodies (MBs). The PB consists of 16 glomeruli arranged in the shape of a handlebar. The FB is located anteroventrally and forms the largest neuropil of the CX. Within the FB, neuronal trajectories are organized to form substructures of horizontal strata and vertical subdivisions. Immediately anterior to the FB lies the EB, a neuropil that is structured into radial sectors and concentric zones. Whereas the PB, FB and EB are midline-spanning neuropils, the ventral-most modules, the NO, are paired. Two further pairs of modules are closely associated with the CX: the bulbs (BUs) and the lateral accessory lobes (LALs).

**Fig. 2.**
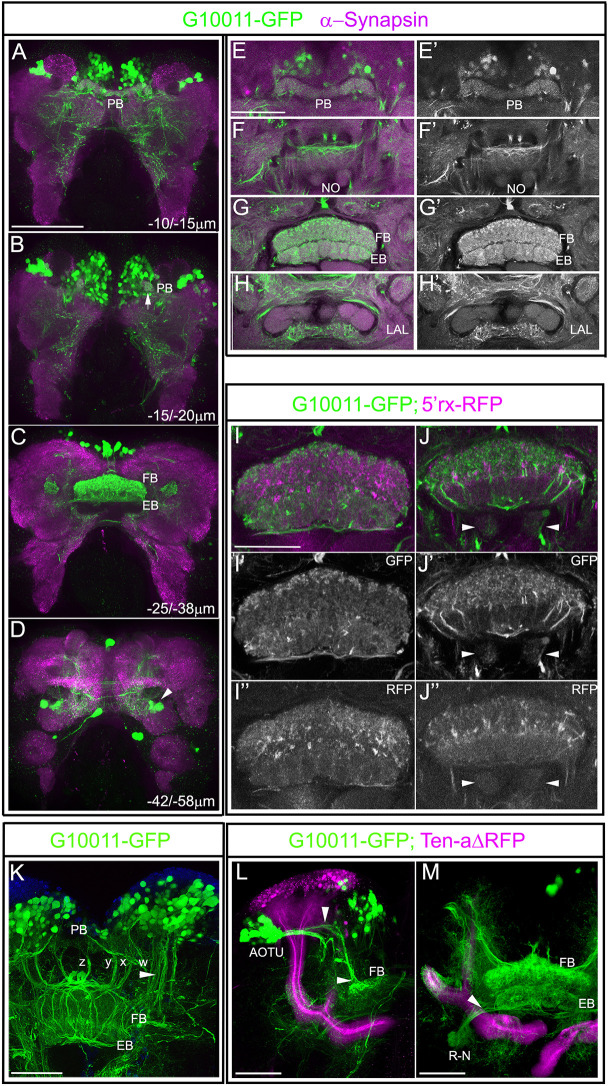
**GFP-labeled CX neuropils in the adult G10011 brain.** Serial confocal sections were combined and visualized as maximum intensity projections to display individual anatomical features. (A-H′) Adult G10011 brains (green indicates GFP autofluorescence) stained with α-Synapsin antibody (magenta). (A-D) Whole brain imaged at low magnification. Scan direction is from the n-D to the n-V surface of the brain. Depth along the *z*-axis is given in µm. (A,B) GFP label within the PB. Arrow in B indicates the most-lateral glomerulus of the PB. (C) GFP label within the FB and EB. (D) GFP label of a subset of ring neurons (arrowhead). (E-H′) Close-ups of CX neuropils in the PB (E), NO (F), FB and EB (G) and LAL (H) regions. We interpret compartments that are located posterolateral to the EB as the LALs. E′-H′ show GFP autofluorescence only. The NO and putative LALs are only weakly labeled by GFP. (I-J″) CX of an animal with the genotype G10011-GFP;5′rx-RFP. I-I″ and J-J″ show two different planes of the CX along the dorsoventral axis. I′,J′ show GFP autofluorescence only. I″,J″ show RFP autofluorescence only. There is little overlap of GFP and RFP fluorescence. Arrowheads indicate the NO. (K-M) Identified sets of neurons with projections into the FB and/or EB. (K) Small-field, columnar neurons arborize within the PB, form the characteristic z, y, x and w fascicles, decussate in the upper part of the FB and establish the columnar organization of the FB. Blue stain is DAPI. (L,M) Adult brain of an animal with the genotype G10011-GFP;Ten-aΔ-RFP. (L) AOTU large-field neurons and their projections into the FB (arrowhead). (M) Large-field ring neurons (R-N) project into the EB body (arrowheads). Scale bars: 100 μm (A-D); 50 μm (E-M). n-D, n-dorsal; n-V, n-ventral.

Neurons, the projections of which make up the neuropils of the CX, are classified as either small-field or large-field neurons (Hanesch et al., 1989; Young and Armstrong, 2010; Yu et al., 2013; Wolff et al., 2015). A well-studied group of small-field neurons are the columnar neurons. These form eight sets of isomorphic cells within each brain hemisphere, and their somata reside within the pars intercerebralis (PI). The projections of a subgroup of columnar neurons form dendritic tufts giving rise to the 16 glomeruli of the PB. Further anteroventrally, columnar neurons project four prominent bilateral pairs of fiber bundles (w, x, y and z tracts). These tracts connect the PB to the FB by interhemispheric crossings before they extend further anteroventrally to establish the columnar structure of the FB. Large-field neurons provide input from other brain areas into the core of the CX. Some large-field neurons project perpendicular to the columnar neuron tracts and effect the horizontal stratification of the FB. For example, such a projection pattern is characteristic for a subset of neurons of the anterior optic tubercle (AOTU): they project first medially and then ventrally to innervate the uppermost stratum of the FB. Another well-studied group of large-field neurons are the ring neurons, the somata of which reside ventrolaterally to the CX and the trajectories of which innervate the EB.

The architecture of the adult CX and its internal connectivity have been well documented in several insect species (Loesel et al., 2002). By contrast, little is known about the regulatory mechanisms that specify subtypes of CX neurons. A few studies have addressed the roles of spatial and temporal factors in the specification of *Drosophila* CX neurons. For example, ring neurons arise from within a spot of *engrailed*-expressing procephalic neuroectoderm and loss of Engrailed results in the loss of embryonic ring neurons (Bridi et al., 2019). Recently, the temporal transcription factor Eyeless and its target, Twin of Eyeless, were shown to specify features of a subset of CX neurons (Sullivan et al., 2019).

Although the overall architecture of the CX is well conserved across different insect species, the size and shape of its neuropils vary, reflecting evolutionary adaptations (Loesel et al., 2002; Strausfeld, 1999; Immonen et al., 2017). We study the regulatory mechanisms that underlie CX development in the red flour beetle *Tribolium castaneum* (He et al., 2019; Farnworth et al., 2020). *Tribolium* is an insect model well suited to the study of gene regulatory pathways: its genome is fully sequenced (Herndon et al., 2020) and *Tribolium* is amenable to genetic manipulation, including enhancer trapping (Trauner et al., 2009). Additionally, ‘parental RNA interference’ (RNAi) is well established as a means to study gene function (Bucher et al., 2002; Schmitt-Engel et al., 2015). General features of embryonic neurogenesis are remarkably well conserved between *Tribolium* and *Drosophil*a (Wheeler, 2003; Biffar and Stollewerk, 2014).

Here, we report a role for the transcription factor Skh, the *Tribolium* ortholog of *Caenorhabditis elegans* uncoordinated-42 (UNC-42), in the specification of a subset of CX neurons. The developmental dynamics of *skh* expression are characteristic for terminal selectors of neuronal subtype identity. *skh* is not expressed in neural progenitors or glia but is expressed in neurons. Many of these neurons survive to adulthood and a subset contributes to the adult CX. Expression of *skh* in CX neurons is maintained into adulthood. Notably, *skh* is absent from neurons that make up other major compartments of the brain. *skh* RNAi results in severe axon outgrowth defects, preventing the formation of an embryonic CX primordium. In addition, we observe a moderate reduction in *skh* expression. The *Drosophila skh* ortholog shows a highly similar expression pattern in the embryo, suggesting a conserved role in the specification of CX neurons.

## RESULTS

### The enhancer trap line G10011 labels several neuropils of the *Tribolium* adult CX

To identify genes that play a role in CX development, we screened a collection of enhancer trap lines that express an untagged version of eGFP (Trauner et al., 2009). Analysis of eGFP fluorescence in *Tribolium* adult brains led to the identification of the G10011 line, in which the CX is heavily labeled (for a 3D image, see Movie 1). G10011 beetles are homozygous viable and fertile, and their lifespan is comparable to that of the wild-type strain *SB* (www.geku-base.uni-goettingen.de). To put G10011-GFP expression into context, we crossed G10011 to Ten-a-Δ-RFP-expressing beetles and examined the adult brains of the resulting progeny. Ten-a-Δ-RFP is a derivative of the enhancer trap line Tenascin-a (also called Teneurin-a)-GFP (He et al., 2019). In Ten-a-Δ adult central brains, RFP expression was restricted to the MBs, which provide a landmark (Fig. 1A-C). Double-labeled brains showed GFP fluorescence in the PB (Fig. 1A, arrowhead), FB and EB (Fig. 1B; for a schematic of CX neuropils and coordinates of the axes, refer to Fig. 1E and 1G, respectively). G10011-GFP-positive somata reside almost exclusively in the posterior brain. The majority of GFP-positive cell bodies were located in the dorsomedial region, where they formed several large clusters within the PI and also more ventral areas. Small clusters of large cells were located in the dorsal-most region of the PI (Fig. 1A,B). These cells projected descending axons, which formed a chiasma in the dorsal brain and then extended further ventrally to enter the VNC. Based on cell body location and axonal projections, these are likely to be neurosecretory cells. In the dorsolateral brain, large clusters of cells resided ventrolaterally to the Kenyon cells (KCs) of the MBs (Fig. 1B, arrowhead). In addition, a small number of GFP-positive cells were scattered throughout the lateral regions of the posterior brain. The anterior cortex of the brain contained only a single GFP-positive cluster comprising six to eight cells, located ventrolaterally to the EB (Fig. 1C, arrowhead). Finally, a few, very large cells within the tritocerebrum projected towards the PI (Fig. 1C, arrow). We determined an average number of 320 GFP-positive cells per brain lobe (*n*=4; Fig. 1F). Notably, G10011-GFP was not expressed in the KCs or antennal lobes. We do not know whether the GFP expression in the optic lobes was attributable to the G10011 insertion because the transformation marker 3xP3 itself directs GFP expression in the optic lobes (Berghammer et al., 1999). The VNC showed no GFP-positive somata but contained multiple GFP-positive longitudinal fascicles, which originate in the brain (Fig. 1D).

To examine GFP fluorescence in CX neuropils in more detail, G10011 adult brains were stained with α-Synapsin, which facilitates visualization of brain neuropils (Fig. 2A-H′). Image analysis at both low (Fig. 2C) and high (Fig. 2G,G′) magnification confirmed that the FB and EB were strongly labeled by GFP. Within the FB, GFP-fluorescence was observed in all columns and strata. Within the EB, GFP labeled all radial segments (Fig. 2G,G′). In addition, all 16 glomeruli of the PB were labeled by GFP (Fig. 2A,B,E,E′). In contrast to the strong GFP label within the midline-spanning neuropils, GFP-fluorescence within the NO was very weak (Fig. 2F,F′). The CX modules were associated with additional neuropils, such as the BU and LALs. In *Tribolium*, neither of these compartments have been described as yet. We observed a bilaterally symmetric brain area located anteroventrally to the FB/EB, which we interpret as the LALs. G10011-GFP fluorescence within the putative LALs was weak (Fig. 2H,H′).

Strong GFP fluorescence in the FB and EB raises the question of whether G10011-GFP labels all neuronal projections that make up these neuropils. To address this question, we crossed G10011 beetles with the imaging line 5′*rx* (*retinal homeobox gene*) in which the FB and EB are intensely labeled by RFP (He et al., 2019). Image analysis of the respective progeny revealed that G10011-GFP and 5′*rx*-RFP fluorescence were largely nonoverlapping, indicating that G10011-GFP labels only a subset of structures within the FB and EB (Fig. 2I-J″).

Anatomical studies in a variety of insects have led to the characterization of small- and large-field neurons, the projections of which make up the neuropils of the CX (Hanesch et al., 1989; Young and Armstrong, 2010; Yu et al., 2013). Cell body location, morphology and projections of many CX neurons are conserved among different insect species (Pfeiffer and Homberg, 2014). Based on these criteria, we were able to identify one type of small- and two types of large-field neurons that express G10011-GFP. First, sets of neurons, the cell bodies of which reside within the PI, showed all properties indicative of columnar neurons (Fig. 2K): their trajectories contributed to the dendritic tufts within the glomeruli of the PB and then extended more ventrally to form four characteristic fiber tracts, named the z, y, x and w tracts. These tracts connected the PB to the FB by interhemispheric chiasmata before they extended further ventrally to establish the columnar structure of the FB (Fig. 1K). Second, in the dorsolateral brain, two large clusters of neurons adjacent to the MB calyces projected two major fiber bundles, one of which extended first medially and then ventrally before it entered the uppermost stratum of the FB (Fig. 2L). We interpret these CX neurons as a ventral subset of the AOTU neurons. Third, we identified the ring neurons (R-N) the somata of which reside ventrolaterally to the EB and the projections of which innervate the EB (Fig. 2M).

### G10011-GFP-positive neurons establish the FB primordium

**Fig. 3.**
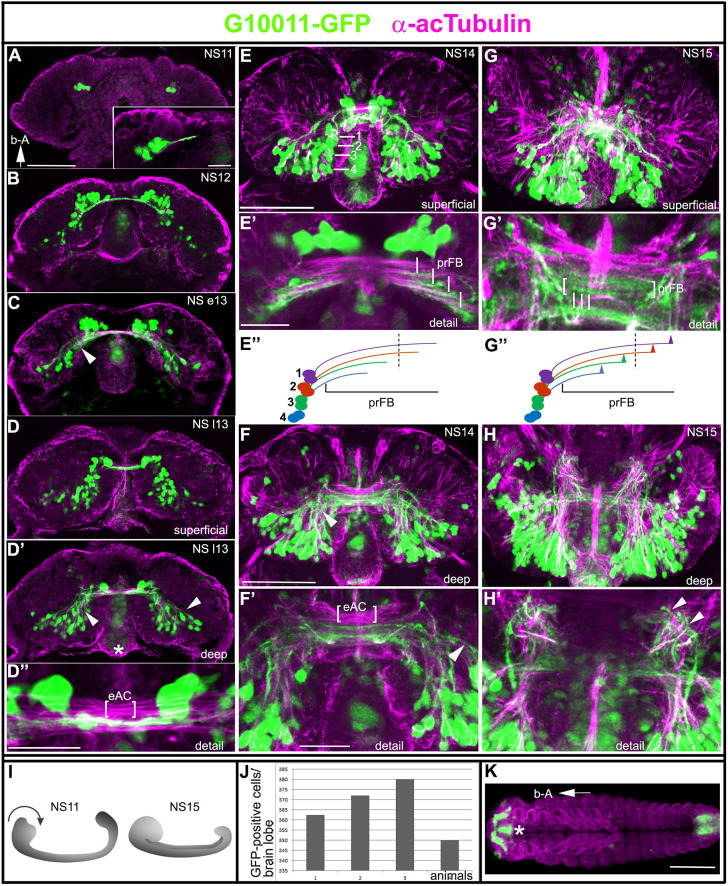
**Embryonic expression of G10011 and the formation of the embryonic commissural system.** (A-H′) Developmental series of G10011-GFP brains from stage NS11 (∼65% embryogenesis) to stage NS15 (100% embryogenesis) (for staging, see Fig. S1). Coordinates are given according to the body axes (b-A arrow in A indicates ‘anterior up’ with respect to the body axis). (A-E′,F-G′,H,H′) Double staining with GFP (green) and acTub (magenta) antibodies. (A-C) Single confocal planes. Inset in A shows the first continuous commissural fascicle, which links both hemispheres of the protocerebrum and is established at late NS11. This primary commissural fascicle is labeled by the GFP antibody. (B) Early born GFP-positive cells are located largely in the anteromedial brain. (C) Multiple GFP-positive cell clusters project their axons from posterior dorsomedial regions towards the primary brain commissure (arrowhead). (D,D′) Serial confocal sections were combined and visualized as maximum intensity projections to depict either superficial (D) or deep-lying (D′) regions of a late NS13 brain. D′ shows multiple GFP-positive cell clusters projecting axons from posterior dorsomedial and dorsolateral regions towards the primary brain commissure (arrows). GFP also labels the stomodeum (asterisk). (D″) Close-up of D′. Multiple commissural fascicles have formed, only a subset of which is GFP positive. The embryonic anterior commissure (eAC) provides a landmark for the position of the prFB within the early commissural system. (E-H′) Serial confocal sections were combined and visualized as maximum intensity projections to depict superficial (E,E′) or deep-lying (F,F′) regions of an NS14 brain. Nearly all GFP-positive cell bodies are located in the dorsoposterior brain. (E) White lines indicate four clusters of cells. We interpret these cells as the progeny of DM1-DM4 that differentiate into columnar neurons. (E′) The four clusters of neurons produce four parallel-running GFP-positive fascicles, which enter the commissural fiber system (vertical white lines). We interpret these fibers as the precursors of the w, x, y and z tracts and, hence, as the prFB. (E″) The trajectories of four cell clusters generate the prFB (1, z tract; 2, x tract; 3, y tract; 4, w tract); dashed line indicates the ventral midline. (F,F′) GFP-positive input into the primary commissure stems largely from cells located in posterior dorsomedial and dorsolateral regions of the brain (arrowhead). The eAC provides a landmark for the position of the prFB within the early commissural system. (G,G′,H,H′) Superficial (G,G′) and deep-lying (H,H′) regions of the NS15 brain. (G) Most late-born GFP-positive cells are found in the posteromedial region of the late embryonic brain. Start of the defasciculation of GFP-positive commissural fiber tracts (white lines). (G′) Note the beginning defasciculation of GFP-positive commissural fiber tracts (white lines indicate individual defasciculating axon tracts). The region of the developing prFB is indicated by brackets. (G″) Schematic of the start of defasciculation; dashed line indicates the ventral midline. (H,H′) Multiple GFP-positive fibers exit the brain and project towards the VNC (arrowheads). (I) Schematic of morphogenetic head movements during embryogenesis. The black arrow indicates the direction of the head involution. (J) Quantification of GFP-positive cells in late-stage NS15 brains. The number of G10011-GFP-positive cells is 362 per brain lobe (mean of four animals, 1-4). (K) Dorsal view of a whole-mount NS14 animal. Embryonic G10011-GFP expression is restricted to the brain, stomodeum (asterisk) and hindgut. Blue stain is DAPI. ‘b-A’ arrow indicates that the anterior is on the left. Scale bars: 50 μm (A-D′,E,F,G,H); 10 μm (inset); 20 μm (E',F',G',H'); 200 μm (K). eAC, embryonic anterior commissure.

CX neurons of holometabolous insects are born during embryonic and larval stages, whereas much of the CX connectivity is established in the pupa. We examined the appearance of G10011-GFP-labeled cells and the establishment of early CX connectivity in the embryonic, larval and pupal stages of *Tribolium* development. First, we addressed G10011-GFP expression during embryogenesis. We observed the embryo staging nomenclature as suggested by Biffar and Stollewerk (2014) which distinguishes 15 stages of neurogenesis: NS1 (0%) to NS15 (100% neurogenesis) (Fig. S1). The earliest expression of G10011-GFP occurred at NS11 (65% of embryogenesis) in two small clusters of cells in anteromedial positions in the brain (Fig. 3A). Subsequently, cell numbers within these clusters increased and additional clusters formed adjacently in more-lateral positions (NS12; Fig. 3B). In addition, clusters of GFP-positive cells appeared in posteromedial regions. Post-NS12, no significant increase in GFP-positive cells in the anteromedial region had occurred. By contrast, in posteromedial and posterolateral regions, multiple new GFP-positive cell clusters arose and early-born clusters gained in cell numbers (Fig. 3C-D′,E,F,G,H). The strongest increase in GFP-positive cells was observed during NS15. At the end of embryogenesis, each brain lobe contained an average of 362 GFP-positive cells (*n*=4; Fig. 2J), the vast majority of which resided in the medial area of the dorsoposterior brain. Embryonic expression of G10011 outside of the brain was restricted to the stomodeum and the hindgut, and GFP-expressing cell bodies were absent from the VNC (Fig. 3K).

In the adult G10011 brain, the columnar neurons of the FB were heavily labeled by GFP (Fig. 2K). We asked whether these cells were of embryonic origin and established the FB primordium (prFB) of the embryonic brain. The prFB is formed by four contralaterally projecting fiber tracts, which emanate from each brain hemisphere and constitute a part of the early commissural system (Farnworth et al., 2020) (Fig. 3E″). These fiber tracts are produced by the progeny of four distinct neuronal neuroblasts (DM1-DM4) located in the posteromedial brain (Andrade et al., 2019; Farnworth et al., 2020). To visualize the development of the commissural system, we co-stained G10011 embryos with α-acetylated Tubulin (acTub). At late-stage NS11, the first acTub-positive fascicle extended towards the midline. This fascicle was also labeled by G10011-GFP (Fig. 3A, inset). From NS12 onwards, GFP-positive fiber tracts made numerous contributions to the commissural system (Fig. 3B-D″,E,F). In NS14, GFP-positive fiber tracts formed that were indicative of the prFB: four contralaterally projecting fiber tracts entered the commissural system as parallel tracts (Fig. 3E′,F). In late-stage NS15, these fibers showed the characteristic pattern of defasciculation, which initiates the development of the columnar architecture of the FB (Fig. 3G′,G″; Farnworth et al., 2020). In *Drosophila*, it has been shown that the contralaterally projecting fibers that constitute the prFB pass through a channel formed by glial membranes (Andrade et al., 2019). We observed a similar arrangement in the *Tribolium* embryonic brain (Fig. S2A-A″). Recently, we showed that a subset of embryonically born columnar neurons express Rx and establish the prFB (Farnworth et al., 2020). Double staining with anti-GFP and anti-Rx revealed a partial overlap of Rx- and G10011-GFP-expressing neurons (Fig. S3B-B″). Taken together, we conclude that G10011-GFP labels embryonically born columnar neurons that establish the prFB.

In addition to columnar neurons, we identified subsets of AOTU and ring neurons in the G10011-GFP adult brain that contribute to the CX. Studies in *Drosophila* showed that some of these neurons are born in the embryo and persist to adulthood (Lovick et al., 2017; Bridi et al., 2019). Given the lack of specific markers, we were unable to identify the ring neurons in the *Tribolium* embryo. We interpret a group of posterolaterally located cells as AOTU neurons (Fig. S2C; arrow). In addition, in the late NS15 brain we observed GFP-positive cells which – based on morphology and location – we interpret as the same large cells observed in the adult tritocerebrum and the putative neurosecretory cells of the prospective PI (Fig. S2D; arrowhead and arrow, respectively).

The adult VNC contained GFP-positive fascicles that originated in the brain (Fig. 1D). We observed that some fascicles arose during embryonic stages (Fig. 3H,H′). For a more detailed display of the major axon tracts in the late-NS15 brain, see Fig. S3.

### G10011-GFP labels immature CX neuropils in the late *Tribolium* larva

**Fig. 4.**
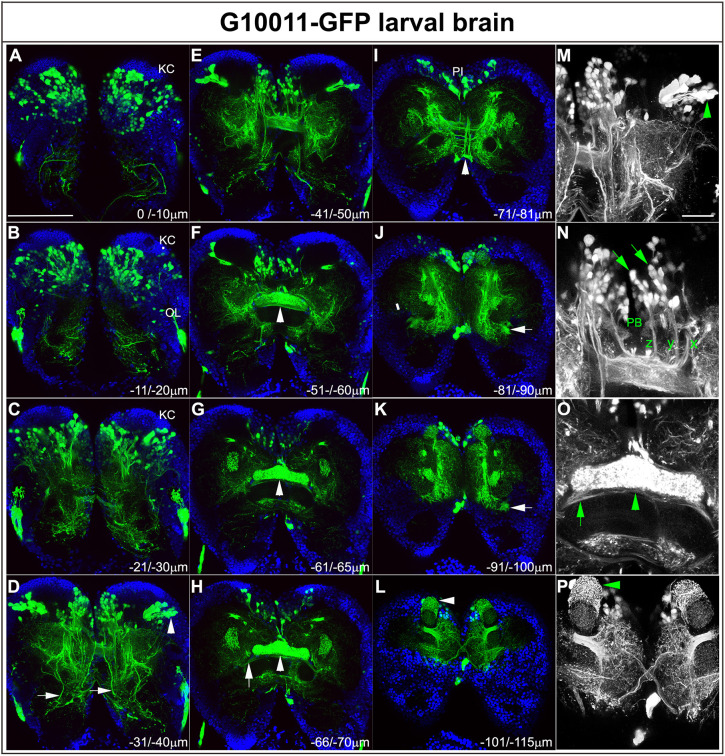
**GFP expression in late G10011 larva.** (A-L) GFP autofluorescence (green) and DAPI (blue) staining. (M-P) GFP only. Serial confocal sections were combined and visualized as maximum intensity projections to display individual anatomical features. Scan direction is from the n-D (A) towards the n-V (L) surface of the brain. Depth along the *z*-axis is given in µm. (A-C) GFP-positive cell bodies in the posterior brain. KCs do not express GFP. GFP fluorescence within the OLs may reflect the expression of the transfection marker 3xP3-GFP. (D,M) Multiple axon tracts originating in the n-anteromedial and n-anterolateral protocerebrum descend towards the VNC (arrows). Arrowheads indicate the dorsal and ventral clusters of AOTU neurons. (E,N) A subset of columnar neurons (arrows) with their arborizations within the PB and their characteristic z, y and x axon tracts (the w tract is not in focus). (F-H,O) The FB is strongly labeled by GFP (arrowheads). Arrows (H,O) indicate axon tracts that originate from the ring neurons and enter the commissural system. Distinct elements of the EB are not yet present. (I) Ascending axon tracts originating from cells of the tritocerebrum (arrowhead) project towards the PI. (J,K) GFP-positive ring neurons (arrows). (L,P) GFP-expressing cells form multiple dendritic arborizations that enwrap distinct parts of the MBs (arrowheads). Scale bars: 100 μm (A-L); 20 μm (M-P). n-D, n-dorsal; n-V, n-ventral.

During larval development, the brain greatly increases in size and undergoes major morphogenetic movements, which together prevent the tracing of individual embryonically born neurons to late larval stages. The number and distribution of GFP-positive cell bodies in the late larval brain (80-90% larval development) closely resembled that of the adult brain (Fig. 4A-L). GFP-positive neurons resided nearly exclusively in the dorsoposterior brain. Most GFP-positive cells were located in the medial brain, with the exception of two large cell clusters, which laterally abutted the KCs (Fig. 4D,M). As in the adult, G10011-GFP expression was absent from the KCs. Location, morphology and, in part, axon trajectories allowed us to recognize sets of cells that we were also able to identify in the adult brain: notably, columnar neurons and a subset of AOTU neurons (Fig. 4D-F), putative neurosecretory neurons of the PI (Fig. 4I,J), a subset of ring neurons (Fig. 4J,K) and the large cells of the tritocerebrum (Fig. 4K-P).

In the late larva, the PB and the FB were clearly labeled by GFP. Fiber tracts emanating from the columnar neurons passed through individual glomeruli of the PB and then extended more ventrally to form tracts with multiple interhemispheric chiasmata before they extended further ventrally to build an immature FB within which a columnar structure was not yet obvious (Fig. 4N). The overall shape of the FB already resembled that of the adult FB but GFP fluorescence showed no obvious dorsoventral stratification at this stage (Fig. 4G,O). In the late larva, fibers projected from the ring neurons to positions immediately ventral of the FB (Fig. 4H,O, arrows). However, no structures characteristic of the pupal/adult EB were detectable. In addition**,** the fibers of the ring neurons did not express Synapsin at this stage (Farnworth et al., 2020). In the late larval brain, each hemisphere contained an average of 384 GFP-positive cells (*n*=4).

### G10011-GFP labels the PB, FB and EB in the late *Tribolium* pupa

**Fig. 5.**
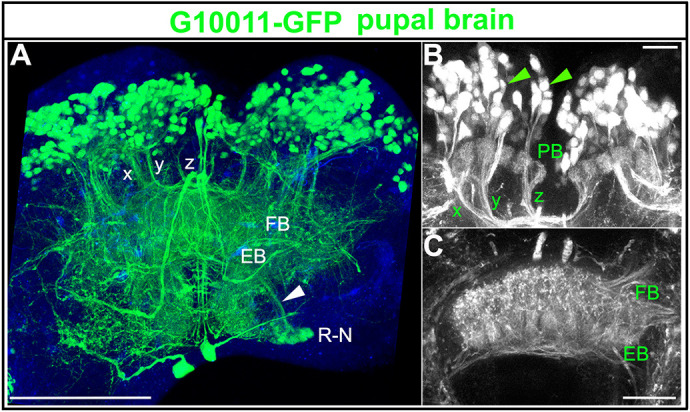
**GFP expression in the late (90% development) G10011 pupal brain.** (A-C) GFP autofluorescence [in A combined with DAPI staining (blue)]. (A) Confocal stack is visualized as a maximum intensity projection. The columnar organization of the FB is well established. The z, y and x axon trajectories of the columnar neurons are indicated (the w tract is not in focus). The ring neurons (R-N) are shown, with their projection towards the EB (arrowhead). (B) Columnar neurons (arrowheads), their arborizations within the glomeruli of the PB and their z, y and x axon trajectories (the w tract is not in focus). The PB is not yet fused at the midline. (C) The EB is well developed in the late pupa. Scale bars: 100 μm (A); 20 μm (B,C).

In the late pupal brain, the number and distribution of GFP-positive cell bodies was almost the same as in the embryonic, larval and adult brain (Fig. 5). The overall architecture of the pupal CX neuropils closely resembled that of their adult counterparts. The glomeruli of the PB were pronounced but fusion at the midline had not yet taken place (Fig. 5B). Within the FB, the columnar structure was well established (Fig. 5A,B). The appearance of the EB with its radial segmentation was in contrast to the late larval brain, in which no EB structures were seen (Fig. 5C).

### G10011-GFP fluorescence reflects the RNA expression of the transcription factor TC-UNC-42

**Fig. 6.**
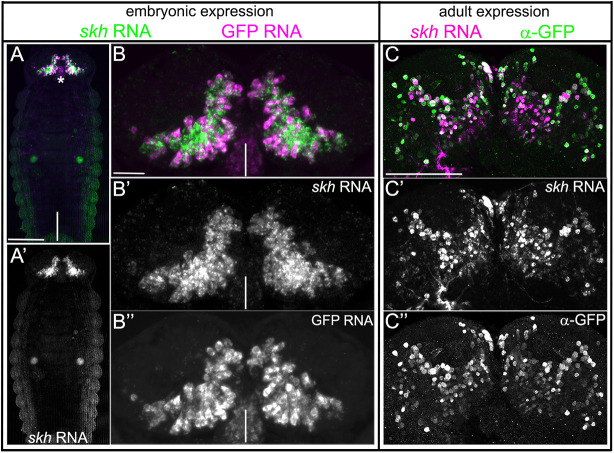
**G10011-GFP reflects the RNA expression of *skh* (TC007335).** (A-B″) Double-fluorescence *in situ* hybridization with a GFP (magenta) and a *skh* (green) RNA probe in an NS14 embryo. (A,A′) Dorsal view of a whole-mount embryo. The expression of GFP and *skh* is restricted to the brain and stomodeum (asterisk). (B,B″) GFP and *skh* RNA expression colocalize in the embryonic brain. Vertical white lines indicate the midline. (C,C″) *skh* RNA *in situ* hybridization (magenta) combined with GFP antibody staining (green) in an adult G10011 brain. Serial confocal sections were combined and visualized as maximum intensity projections. *skh* RNA and GFP protein are colocalized. For additional images, see Figs S4 and S5. Scale bars: 100 μm (A,A′,C-C″); 20 μm (B-B″).

We mapped the plasmid insertion site of G10011 to the genomic position 6024777 within the first intron of *TC008169* (for details, see Fig. S4A-B‴). However, the expression of this gene did not match G10011 GFP fluorescence. Another candidate gene in this region is *TC007335*, the putative transcriptional start site of which is located 18.5 kb upstream of the insertion site. To examine whether G10011-GFP reflects the expression of *TC007335* in the embryo, we performed double fluorescence *in situ* hybridization with a GFP and a *TC007335* RNA probe. The GFP and *TC007335* signals colocalized at all embryonic stages, indicating that G10011-GFP faithfully reports *TC007335* expression (Fig. 6A-B″; Figs S4 and S5). Furthermore, *TC007335* RNA *in situ* confirmed that expression is restricted to the brain and stomodeum (Fig. S4C). We named *TC007335 shaking hands* (*skh*) to highlight the chiasma formed by cells of the PI (Fig. 1B). *skh* encodes the ortholog of the *C. elegans* transcription factor UNC-42, a PRD-like homeodomain protein (Baran et al., 1999) (for a phylogenetic tree, see Fig. S6A).

G10011-GFP fluorescence was strong in the adult brain. To investigate whether this reflects GFP perdurance or the continued expression of *skh*, we performed *skh* RNA *in situ* staining combined with GFP staining in whole-mount adult brains. Most, and possibly all, *skh* RNA-positive cells were also GFP positive, demonstrating the continued expression of the *skh* transcript (Fig. 6C-C″; Fig. S5). However, because of technical limitations, we cannot exclude the possibility that a small number of *skh* RNA-positive cells do not express GFP.

### *skh* expression is restricted to neurons

**Fig. 7.**
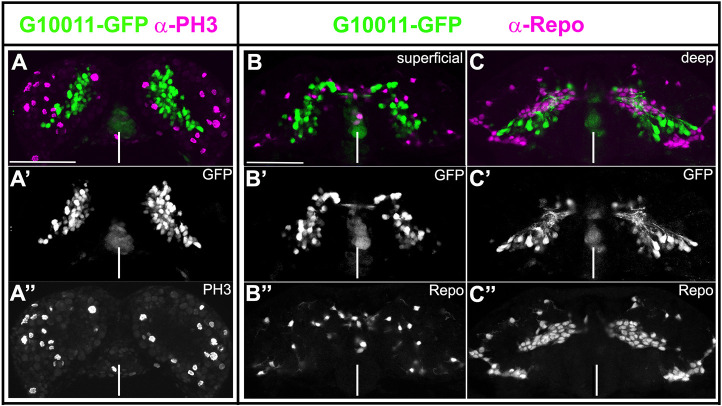
**G10011-GFP expression is restricted to nondividing, nonglial cells.** Embryonic G10011 brains were stained with GFP (green) and PH3 (A-A″) or Repo (B-C″) (both magenta) antibodies. Serial confocal sections were combined and visualized as maximum intensity projections. (A-A″) NS13. (B-C″) NS14. There is no overlap of GFP- and PH3- or Repo-expressing cells. White lines indicate the midline. Scale bars: 50 μm.

Embryonic and larval brains contain mitotically active and postmitotic cells. Mitotically active cells are NBs and their immediate progeny. To determine whether *skh* is expressed in mitotically active cells, we double-stained embryos and larval brains with antibodies against GFP and the mitosis marker Phospho-histone-3 (PH-3). Colocalization of GFP and PH-3 signals was not observed, indicating that *skh* expression was restricted to postmitotic cells in the analyzed stages (Fig. 7A-A″). This conclusion is supported by the observation that GFP fluorescence was absent from the superficial NB layer of the brain. In addition, we double-stained embryos and adult brains with antibodies against GFP and the glial marker Repo. GFP and Repo signals did not colocalize (Fig. 7B-C″). Thus, we conclude that *skh* expression is restricted to neurons in embryonic and adult brains.

### *skh* knockdown results in axon outgrowth defects and a reduction in GFP-positive cells

**Fig. 8.**
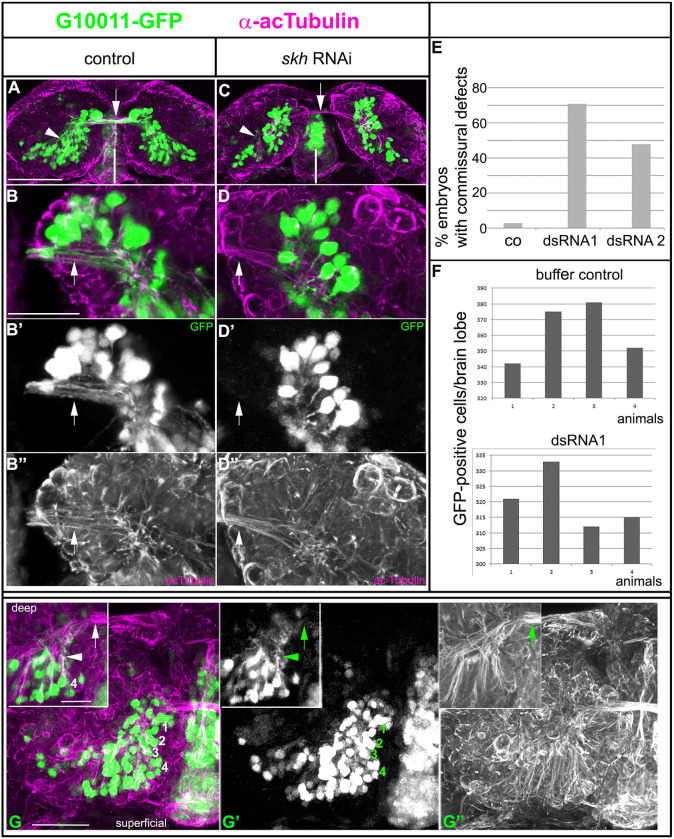
**Parental RNAi of *skh* leads to severe axon outgrowth defects in the embryonic brain.** Dorsal views of G10011-GFP NS14 brains double stained with GFP (green) and acTub (magenta) antibodies. (A-B″) Control brain (progeny of buffer-injected pupae). (A) Whole brain at low magnification; GFP-positive axon tracts join the commissural system linking both hemispheres of the brain (arrow). The arrowhead indicates GFP-positive cell clusters in the posterior brain. The vertical white line indicates the midline. (B-B″) Close-ups; GFP-positive axons project towards the midline. GFP-positive input into the primary commissure (arrow) stems largely from cells located in posterior dorsomedial and dorsolateral regions of the brain. (C-D″) *skh* RNAi brain. (C) Whole brain at low magnification; GFP-positive axons fail to join the commissural system. Arrowhead indicates the loss of GFP-positive cell clusters. Arrow indicates fibers within the commissure. (D-D″) Close-ups; (D′) GFP-positive axons stall, whereas most acTub-positive axons are unaffected (D″). The vertical white line indicates the midline. Arrows indicate fibers within the commissure. (E,F) Quantification of *skh* RNAi phenotypes. (E) Commissural defects were scored at stages NS14 and NS15. Buffer-injected control (co) *n*=80 (two biological replicates) 3% defects; dsRNA frag1 *n*=85 (two biological replicates) 71% defects; and dsRNA frag2 *n*=35, 48% defects. (F) Quantification of the number of GFP-positive cells. The loss of GFP-positive cells in *skh* RNAi embryos was scored at NS15. Buffer-injected control (co), 362 GFP-positive cells (mean of animals 1-4); dsRNA frag1, 308 GFP-positive cells (mean of animals 1-4). (G-G″) Loss of GFP-positive commissural fascicles results largely from fascicle stalling. Superficial layer of a G10011-GFP brain at NS14. There is an absence of GFP-positive fibers within the commissure (arrowheads) despite the presence of cell clusters representing the progeny of the neuroblasts DM1-DM4 (labeled 1-4 in G). Insets show a deep layer of the same brain with the fascicle emanating from the DM4-derived cell cluster stalling (arrowheads). Arrows indicate fibers within the commissure. Original stacks can be viewed at https://figshare.com/projects/Additional_data_for_Garc_a_P_rez_et_al_Tribolium_shaking_hands_is_a_putative_terminal_selector_and_controls_axon_outgrowth_of_central_complex_neurons/93149. Scale bars: 50 μm (A,C); 20 μm (B,D″); 10 μm (insets).

To explore the effects of reduced Skh function in the embryo, we performed parental RNAi in G10011 animals using two nonoverlapping double-strand (ds)RNA fragments (‘frag1’ and ‘frag2’). Knockdown phenotypes were examined by double staining with GFP and acTub antibodies. Loss of Skh had drastic consequences for the outgrowth of all G10011-GFP-positive axons: in severely affected embryos, no contralaterally projecting axons entered the commissural system and, hence, no prFB formed (Fig. 8). Axon outgrowth defects occurred with high penetrance: RNAi with ‘frag1’ and ‘frag2’ resulted in severe defects in 71% and 48% of the embryos, respectively. Examination of GFP-fluorescent cells showed that some axon outgrowth still took place but axons terminated prematurely close to the respective cell bodies (Fig. 8D). Axon outgrowth defects were observed in all GFP-expressing neurons, including the progeny of DM1-DM4, which, in wild type, generate the prFB (Fig. 8G-G″). Axon extension defects were restricted to GFP-positive trajectories: acTub-positive but GFP-negative axon trajectories formed normally (Fig. 8C,D″,G″). These results suggest that the requirement for Skh in axon extension is cell-autonomous.

In addition to axonal defects, we observed a moderate reduction in GFP-positive cells in knockdown embryos (compare Fig. 8A with Fig. 8C; quantification in Fig. 8F). Given the lack of specific markers for G10011-GFP-positive cells, we were unable to determine whether loss of GFP was due to apoptosis or the transformation of cell identity. Loss of GFP expression in DM1-DM4 progeny occurred with low penetrance and, hence, is unlikely to be the main cause of the loss of GFP-positive commissural fibers, which constitute the prCX (of nine embryos with no GFP-positive commissural fibers, only three showed loss of one or more GFP-positive DM1-DM4 clusters; one example is shown in Fig. 8G).

*Skh* RNAi animals did not develop to late larval stages, preventing analysis of postembryonic CX defects.

### The embryonic expression patterns of *Tribolium* and *Drosophila skh* are conserved

**Fig. 9.**
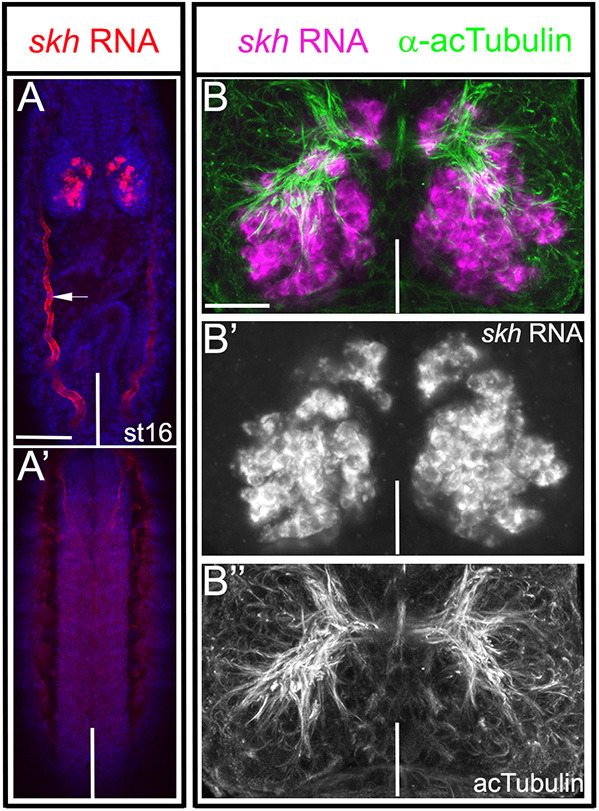
**Embryonic expression of *skh* in *Drosophila*.** (A,A′) *skh* RNA *in situ* hybridization (red) and DAPI staining (blue) of stage-16 whole-mount embryo, with the anterior facing up. (A) Dorsal view; (A′) ventral view. In *Drosophila*, *skh* RNA is restricted to the brain. Red fluorescence in the trachea is an *in situ* artifact (arrow). (B) Spatial organization of *skh* RNA-expressing cells and major axon tracts showing *skh* RNA *in situ* hybridization (magenta) combined with acTub (green) immunostaining. (B′) *skh* RNA only. Comparison with Fig. 6B shows that embryonic *skh*-expressing cells are similarly distributed in *Tribolium* and *Drosophila*. (B″) acTub immunostaining only. Vertical white lines indicate the dorsal midline in all panels except A′, in which it marks the ventral midline. Scale bars: 50 μm (A,A′); 20 μm (B-B″).

The *Drosophila* ortholog of *Tribolium skh* is encoded by CG32532 (Fig. S6). Its gene product is a homeodomain protein, which remains uncharacterized. We examined the embryonic expression pattern of *Dm*-*skh* by RNA *in situ* hybridization (Fig. 9A-B). As seen for its *Tribolium* ortholog, in *Drosophila*, *skh* was expressed in the brain but was absent from the VNC and non-neural tissues (Fig. 9A,A′).

To compare the spatial arrangement of *Drosophila* and *Tribolium skh*-positive cells, we double-labeled *Drosophila* embryos with *skh* RNA and acTub antibody and examined the positions of cell bodies relative to the commissural system. At the end of embryogenesis, the spatial arrangement appeared highly similar in both organisms (compare Fig. 9B-B″ with Fig. 6B′): small clusters of *skh*-positive cells were located immediately anteriorly to the commissural system, whereas the vast majority of cells resided posteriorly to the commissural system in the dorsomedial brain. In a few cases, we were able to follow the trajectories of *skh*-positive cells and found that some entered the commissural system (Fig. 9B-B″).

## DISCUSSION

### G10011-GFP is a useful tool for the study of the dynamics of CX development

Although the anatomy of the adult CX has been well described in many insect species, the CX is vastly understudied from a comparative developmental perspective. Other than a large body of work addressing CX development in *Drosophila*, the CX has been investigated in the desert locust (Heinze and Homberg, 2008; Boyan and Williams, 2011; Boyan et al., 2017), monarch butterfly (Heinze et al., 2013) and, more recently, in the dung beetle (Immonen et al., 2017). Developmental studies in non-*Drosophila* models are hampered by not only a lack of anatomical information at the single-cell level, but also a near-complete lack of molecular and genetic tools. We seek to establish *Tribolium* as an alternative insect model in which to study CX development (He et al., 2019; Farnworth et al., 2020). G10011-GFP marks the *Tribolium* CX. As reported previously, the *Tribolium* adult PB is continuous (Dreyer, 2010), a feature that is shared with several other insects, including the dung beetle (Immonen et al., 2017). Moreover, the subdivision of the PB into eight glomeruli in each brain hemisphere is a shared feature of *Tribolium*, the desert locust, monarch butterfly and dung beetle, but not *Drosophila*, which has nine paired glomeruli (Heinze and Homberg, 2008; Heinze et al., 2013; Immonen et al., 2017). As described for the dung beetle, the *Tribolium* EB is sausage shaped and organized in vertical slices (Immonen et al., 2017).

Our results suggest that many GFP-positive CX neurons are born early during development, making G10011-GFP a useful tool for the study of CX formation: G10011-GFP expression confirms and extends the earlier findings that the FB is largely assembled in the larva, whereas a distinct EB forms later in the pupa (Panov, 1959; Koniszewski et al., 2016; Farnworth et al., 2020). In combination with additional markers, G10011-GFP will be a valuable tool to identify and characterize a subset of CX neurons at the single-cell level.

The *Drosophila* ortholog of *skh* shows an RNA pattern in the embryo that is highly similar to that of *skh*, suggesting that early expression is conserved. The generation of a corresponding imaging line in *Drosophila* should provide a means for a comparative study of *Drosophila* and *Triboliu*m CX development at the anatomical level.

### Skh is a putative terminal selector of neuronal subtype identity

Terminal selector expression in neurons is continuous from cell birth to cell death. Therefore, such factors provide excellent markers for subsets of neurons for developmental, molecular and evolutionary studies. With this work, we identify Skh as the first putative terminal selector in neurons that contribute to the CX. Skh is the ortholog of *C. elegans* UNC-42, the role of which in the specification of neuronal subtypes has been well described (Wightman et al., 1997). *unc-42* was first discovered by Brenner in his classic screen of mutants that showed abnormal locomotion (Brenner, 1974). A later study showed that UNC-42 is required for axon pathfinding in a subset of neurons that facilitates a specific locomotor routine (Baran et al., 1999). Studies of *C. elegans* UNC-42 and other transcription factors led to the concept of terminal selectors as regulators of neuronal subtype identity (Hobert, 2008). In contrast to developmental genes that are expressed early in the gene regulatory cascade, terminal selectors are the final targets of the cascade. Maintenance of terminal selector expression is accomplished by positive autoregulatory feedback loops; accordingly, loss of activity results in the loss of its expression at later stages. The lifelong expression of terminal selectors facilitates the regulation of early aspects of subtype differentiation, such as axon pathfinding, as well as late aspects, including the maintenance of structural and molecular features of the mature neuron.

Our study shows that *Tribolium skh* expression is characteristic for terminal selector genes. We hypothesize that the adult expression of *skh* reflects the lifelong expression in many embryonically born neurons; however, because of the lack of genetic tools for permanent cell marking, we can demonstrate this only for embryonically born columnar neurons and neurons of the PI that can be traced to adulthood. An early aspect of columnar neuron identity is their axonal projection, which leads to the establishment of the prFB. Knock down of Skh abolishes axon outgrowth, indicating that a requirement of Skh for the development of connectivity is conserved between *Tribolium* and *C. elegans*. In late *Tribolium* knockdown embryos, we observe a moderate loss of G10011-GFP fluorescence, suggesting that maintenance of *skh* expression by an autoregulatory feedback loop may be another conserved feature.

The term ‘terminal selector’ derives from studies in *C. elegans* in which some transcription factors directly co-regulate differentiation genes, which together bring about all the specific features of a distinct neuronal subtype. The target genes of *skh* are currently unknown. During early development, they are likely to include differentiation genes required for axon pathfinding. The question of whether *skh* coordinately directs the expression of a battery of effector genes at any developmental stage remains to be investigated.

*skh* expression is not restricted to one particular neuronal subtype but is found in many neurons with different morphological features. Therefore, we expect additional transcription factors to act in parallel to, or in combination with, Skh to specify distinct identities.

Comparing the expression patterns and the Skh target genes in *Tribolium* and *Drosophila* CX neurons will contribute to a better understanding of CX formation and may uncover a molecular basis for anatomical differences of the CX in these species.

## MATERIALS AND METHODS

### Animal husbandry

*T. castaneum* (NCBI: txid7070) beetles were maintained on standard wholemeal wheat flour (type 1050) at 28°C. To obtain embryos, the beetles were transferred to fine wheat flour (type 405) and kept at 32°C. Egg laying was allowed for 24 h. Subsequently, the embryos were separated from the beetles, aged for an additional 24-48 h at 32°C and then collected for fixation. The San Bernadino strain was used as wild type.

*D. melanogaster* Oregon R flies were maintained at 18°C on standard cornmeal agar supplemented with dry yeast flakes. To obtain embryos, flies were placed in collection cages with apple juice-agar plates smeared with fresh yeast paste and placed at 25°C. Egg laying was allowed for 4 h. Then, the apple juice plates were removed from the cages, aged for an additional 16 h at 25°C and then collected for fixation.

### Fixation

*Tribolium* embryos were collected, fixed and stored as previously described (Buescher et al., 2020). *Tribolium* larval, pupal and adult brains were dissected in ice-cold 1× PBS for up to 30 min. Then, methanol-free formaldehyde was added to a final concentration of 4% (v/v). Fixation was performed for 30, 45 or 60 min on ice for larval, pupal and adult brains, respectively. Subsequently, the brains were washed three times for 20 min each with ice-cold 1× PBST (PBS including 0.1% Triton X-100; Sigma-Aldrich). In the second wash, DAPI was added to a final concentration of 1 ng/µl. Brains not dedicated to immunohistochemistry were mounted in VectaShield H-1000 (Vector Laboratories) and imaged immediately. Brains dedicated to immunohistochemistry were placed into blocking solution containing 3% (w/v) bovine serum albumin (BSA; fraction V, Roth) and 0.05% sodium azide (Sigma-Aldrich). Adult brains dedicated to RNA *in situ* hybridization were dehydrated by putting them through an ethanol (ETOH) series: 25% ETOH:75% PBS, 50% ETOH:50% PBS and 75% ETOH:25% PBS for 5 min incubation each. Finally, the brains were placed in 100% ETOH and kept at −20°C for several days prior to *in situ* hybridization.

### Immunohistochemistry

*Tribolium* and *Drosophila* embryos: methanol was discarded from the fixed embryo collections. Subsequently, the embryos were washed three times with 1× PBST for 20 min each at room temperature (RT). Embryos were blocked for 1 to 2 h in 3% BSA (w/v) (containing 0.05% sodium azide) at RT. Primary antibodies were added at the indicated concentrations (Table S1) and incubation was performed overnight on a rotating wheel at 4°C. The embryos were then washed three times with 1× PBST for 30 min each at RT to remove the primary antibodies. Secondary antibodies (Table S1) were added at a dilution of 1:1000 and incubation was performed on a rotating wheel for 90 min at RT. Subsequently, the embryos were washed three times for 20 min each with 1× PBST. In the first wash, DAPI was added to a final concentration of 1 ng/µl. Finally, as much liquid as possible was removed and VectaShield mounting medium was added. *Tribolium* germ bands were freed of yolk with the help of a fine brush, mounted with the dorsal side up and imaged. *Drosophila* embryos were pipetted onto microscope slides and imaged as whole mounts.

Immunohistochemistry with adult brains was performed essentially as for the embryos except for the following modifications: the concentration of Triton X-100 was increased to 0.5%, the incubation period with the primary antibody was extended to ∼40 h and incubation with the secondary antibody was performed overnight at 4°C.

See Table S1 for a list of all primary and secondary antibodies used in this study.

### FISH

Single and double fluorescence RNA *in situ* hybridization and RNA *in situ* hybridization followed by antibody staining of *Tribolium/Drosophila* embryos was performed as previously described (Buescher et al., 2020). To generate a *skh*-specific RNA *in situ* probe, a DNA fragment was generated by PCR using wild-type embryonic cDNA (*Tribolium/Drosophila*) as templates (for details, see Table S1). The PCR products were cleaned by gel-electrophoresis, extracted and used as templates for an additional round of amplification using the same gene-specific primer pairs but with the modification of an added T7 RNA transcriptase-binding site at the 5′-end of the reverse primer. Digoxigenin- or fluorescein-labeled RNA probes were produced using a Roche RNA labeling kit. For RNA *in situ* hybridizations, the probes were used at a concentration of 4 ng/µl (total hybridization volume: 50-100 µl).

RNA *in situ* hybridization in *Tribolium* adult brains was performed essentially as for embryos, except for the following modifications: the concentration of Triton X-100 was increased to 0.5%, the RNA hybridization period was prolonged to 48 h and incubation with the respective antibodies was performed for 48 h at 4°C.

### Image acquisition

Confocal serial scanning images were acquired at 1.5-2 µm intervals using a LSM 510 microscope (Zeiss) using either a 20×0.5 Plan-Neofluar or a 40×1.4 Plan-Neofluar objective (Zeiss). Stacks were processed using the Zeiss LSM Browser software and whole or parts of stacks were visualized as maximum intensity projections. Brightness, contrast, size and resolution of the images were processed in Adobe Photoshop CS. The movie of the G10011 adult brain was generated with ImageJ software (Movie 1).

### Nomenclature used in anatomical analysis

For *Tribolium* and *Drosophila* embryos, the axes used for anatomical analysis in this study were the body axes (in Fig. 3A,K, ‘b-A’ indicates anterior with respect to the body axis). For *Tribolium* postembryonic stages (larva, pupa and adult), the axes used for anatomical studies were neuraxes. According to the neuraxes, the protocerebral bridge and the fan-shaped body are located n-dorsal of the ellipsoid body. For a detailed description of the body axes and the neuraxes in *Tribolium* and *Drosophila*, refer to Farnworth et al. (2020).

### Insertion site mapping of the enhancer trap line G10011

The genomic location of the plasmid insertion was determined by inverse PCR (Thibault et al., 2004). Genomic DNA was extracted from three beetles following the protocol in Thibault et al. (2004). The genomic DNA was digested with the restriction enzyme Sau3AI, highly diluted and ligated under conditions that facilitated intramolecular circularization. Subsequently, the ligation products were amplified by PCR using plasmid-specific primers. The PCR product was cleaned up by gel electrophoresis, extracted and sequenced. Blasting of the sequence against the *Tribolium* genome (genome release 3.0) indicated the insertion of the plasmid on chromosome 4 at the genomic position 6024777, which is 18.5 kb upstream of the predicted gene *TC007335* (transcription start site 6006266). Using double fluorescence *in situ* hybridization, we confirmed that the GFP-expression of G10011 reflected the RNA expression of *TC007335* in the embryo and the adult brain. Sequence analysis of the predicted coding region indicated that *TC007335* encodes a paired-like homeodomain transcription factor and is the ortholog of *C. elegans unc-42* and *Drosophila CG32532*. We refer to *TC007335* and *CG32532* as *skh*.

### *skh* knockdown

For parental RNAi in *Tribolium*, 300-400 female G10011 pupae (at 70-80% pupal development) were injected with dsRNA (2 µg/µl) or injection buffer only (control) using a FemtoJet Express (Eppendorf). Injected pupae were placed on fine wheat flour (type 405) for 24 h at 28°C. Eclosed beetles were added to ∼200 male G10011 beetles and maintained for another 24 h at 28°C. Then, all beetles were collected, placed on fresh fine wheat flour and shifted to 32°C. Eggs were collected for 24 h, aged for an additional 48 h and then fixated for immunostaining. Eggs were collected for eight consecutive days. Pupal injections were performed twice with fragment 1 and once with fragment 2 (see following section).

### Generation of gene-specific dsRNA fragments

Embryonic cDNA (0-72 h), prepared from the San Bernadino wild-type strain, was used as template for the generation of gene-specific fragments within the predicted *TC007335* transcribed region. Two primer pairs were used to generate two nonoverlapping fragments (fragment 1, 287 bp; fragment 2, 261 bp) by PCR (for details, see Table S1). The products were cleaned up by gel electrophoresis, extracted and used as templates for an additional round of amplification with the same gene-specific primer pairs but with the modification of added T7 RNA transcriptase-binding sites at both 5′-ends. The PCR products were used as templates for large-scale RNA synthesis using the MEGAscript T7 Transcription Kit (Invitrogen). The dsRNA was precipitated with LiCl, washed with 70% ETOH, dried and dissolved in injection buffer (1.4 mM NaCl, 0.07 mM Na_2_HPO_4_ 0.03 mM KH_2_PO_4_, 4 mM KCl, pH 6.8) to a concentration of 2 µg/µl.

## Supplementary Material

Supplementary information

Reviewer comments
