# Supplementary material for: Shaking hands is a homeodomain transcription factor that controls axon outgrowth of central complex neurons in the insect model Tribolium
Source: Development. 2021 Oct 13;148(19):dev199368. doi: 10.1242/dev.199368 (PMC8543150; doi:10.1242/dev.199368)
Supplement: Supplementary information [file develop-148-199368-s1.pdf]

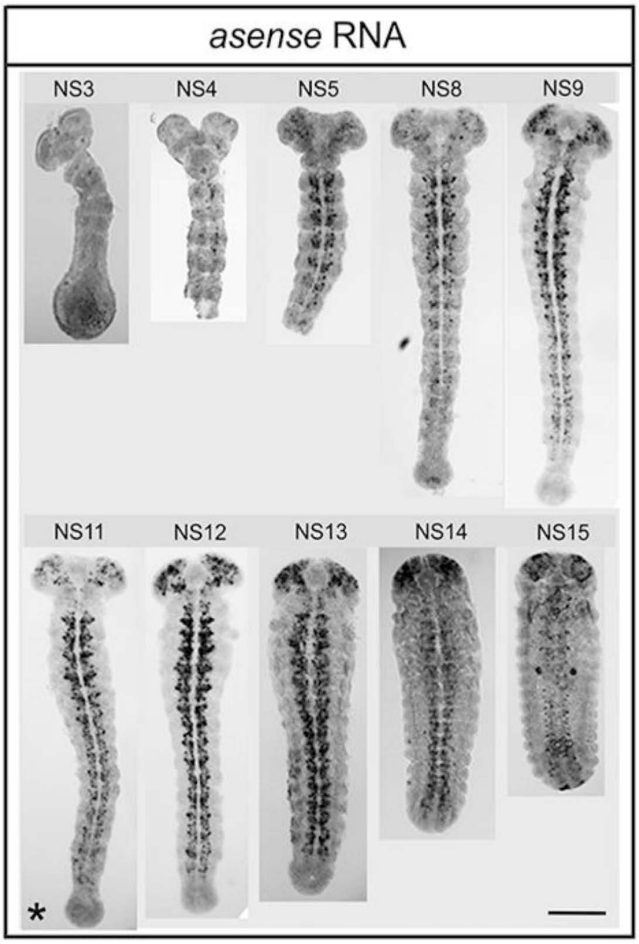

Fig. S1. Embryonic development of *Tribolium*; dorsal view of whole-mount embryos. Neuroblasts (NBs) are visualized by asense RNA in situ hybridization. *Tribolium* embryogenesis is subdivided into 15 stages from NS1 (0% embryogenesis) to NS15 (100% embryogenesis). NB formation in the brain begins at stage NS4. Subsequently, the number of NBs increases steadily until stage NS13/NS14. The precise number of brain NBs that are generated during embryogenesis is not known, but it is estimated to be around 100 NBs per lobe. G10011-GFP-positive cells are first observed at approximately 60% of embryonic development (stage NS11; marked by an asterisk). Scale bar: 200 µm.

Development • Supplementary information

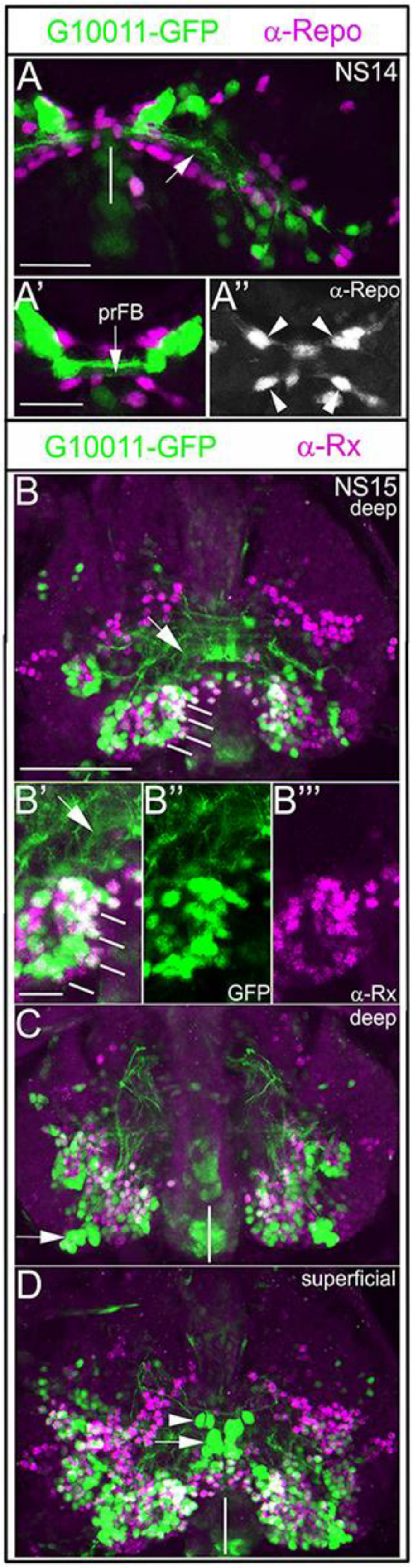

Development • Supplementary information

Fig. S2. Embryonic G10011-GFP-positive neurons establish the FB primordium (prFB). (**A-A''**) Double-immuno-staining with α-GFP (green) and α-Repo antibody (magenta). Cell membranes of midline-associated glia (arrowheads in **A''**) form a channel through which the DM1-DM4 progeny project their trajectories which constitute the prFB (arrows in **A** and **A'**; white line in **A** marks the midline). (B-D) Double-immuno-staining with α-GFP (green) and α-Rx antibody (magenta). (B) The prFB (arrow) is established by the progeny of four neuroblasts [in *Drosophila* called DM1-DM4; white lines (Andrade et al., 2019)]. (B'-B''') Many prFB neurons have been shown to express Rx protein (Farnworth et al.; 2020). A subset of Rx-positive neurons co-expresses G10011-GFP. Arrow in **B'** indicates the trajectories of the progeny of DM1-DM4 . (C) We interpret a group of posterolaterally located cells as AOTU neurons (arrow). (D) We interpret a cluster of very large G10011-GFP-positive cells near the midline (white line) as neurosecretory cells of the prospective PI (arrow). A small cluster of prominent cells is labelled with an arrowhead. In *Drosophila* neurons in this position and with similar morphology and axon trajectories express the neuropeptide HUGIN. Scale bars: (A-A'') 20 µm; (B'-B''') 10 µm; (B,C,D) 50 µm.

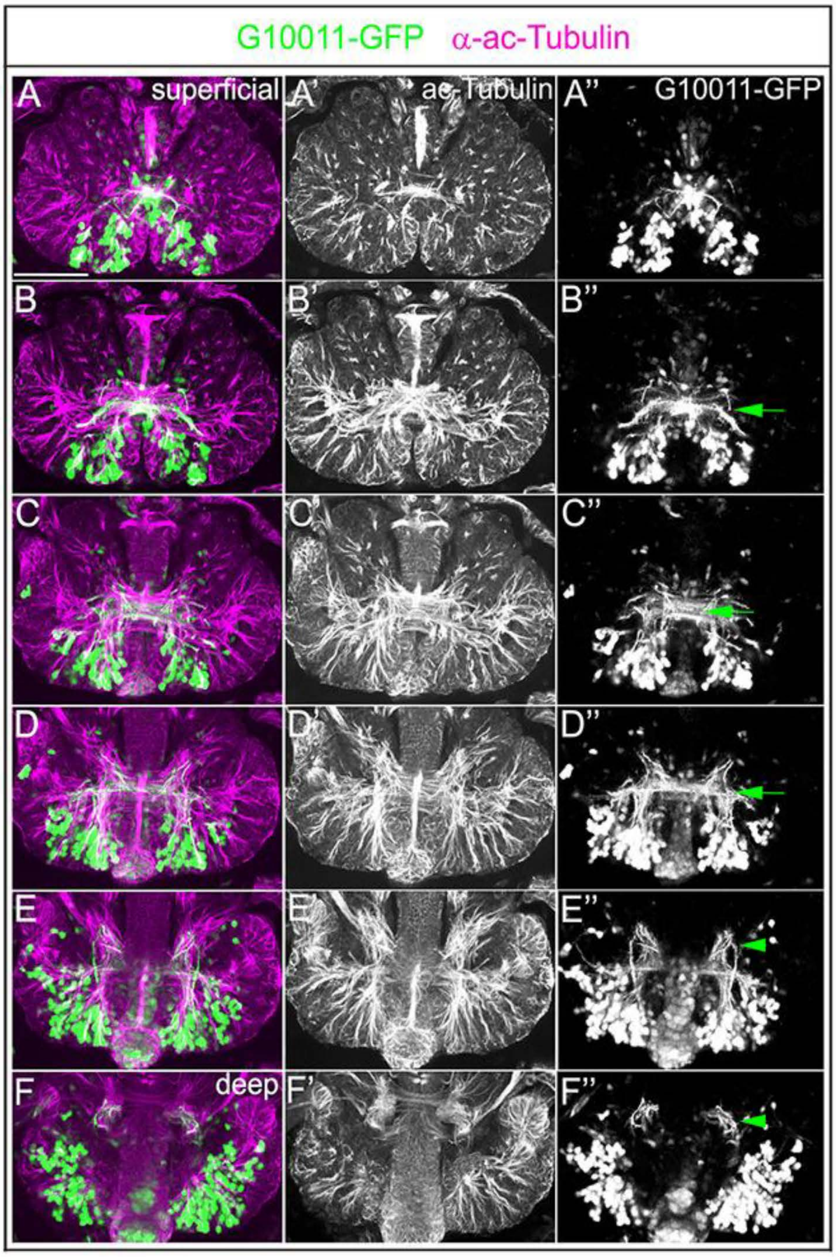

Fig. S3. G10011-GFP positive fascicles contribute to several major axon tracts in the embryonic brain. (A-F**)** G10011 brain at late stage NS15 stained with α-GFP (green) and α-acetylated Tubulin (magenta). Serial confocal sections were combined and visualized as maximum intensity projections to display individual anatomical features. (A'-F') acetylated Tubulin only. (A''-F'') GFP only. (B''-D'') GFP-positive axon trajectories make multiple contributions to the commissural system (arrows). (E'',F'') GFP-positive fibres contribute to longitudinal axon tracts which extend towards the VNC (arrowheads). Scale bar: (A-F'') 50 μm.

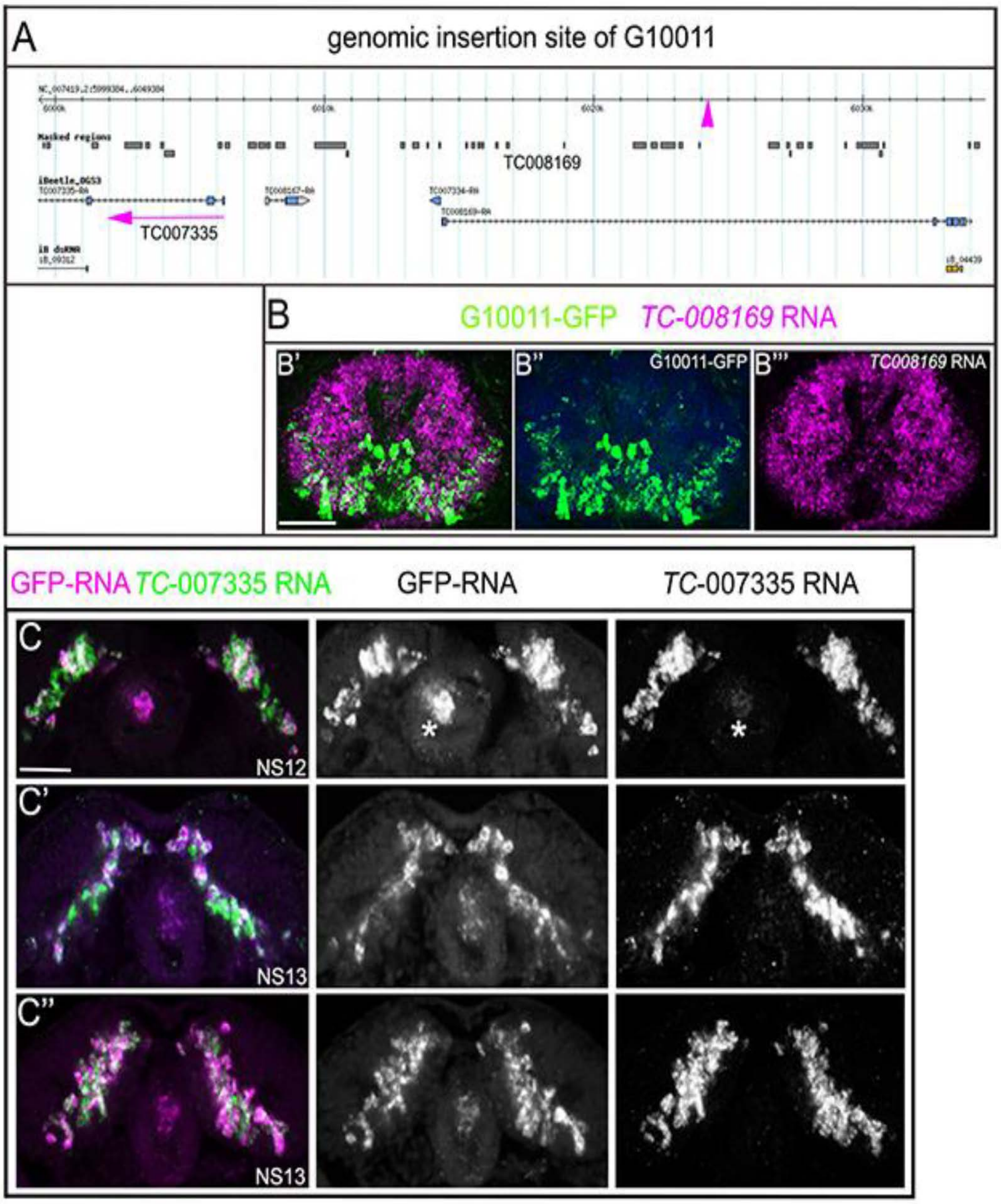

Fig. S4. Identification of the G10011 associated gene. Top panel: (A) Genomic localization of the G10011 plasmid insertion site. The plasmid insertion was mapped to the position 602477 on the fourth chromosome (genome release 3.0). The insertion site is located in the first intron of the predicted gene *TC008169* (magenta arrowhead). We found no

Development • Supplementary information

experimental evidence for the expression of the first *TC008169* exon suggesting that the plasmid insertion site is inter- rather than intragenic. *TC008169* encodes an EF1 hand protein. (B) To determine whether the expression of G10011-GFP reflects the expression of *TC008169*, we performed RNA in situ hybridization with a *TC008169* probe (magenta) combined with a α-GFP staining (green). **(**B”’) *TC008169* expression is pan-neural in the embryonic brain while G10011-GFP is expressed only in a subset of cells (B’’). We conclude that G10011-GFP is unlikely to reflect the expression of *TC008169*. The plasmid insertion site is located 18.5 kb upstream of the predicted gene *TC007335* (transcription start site 6006266, magenta arrow in (A). (C-C’’) The *TC007335* RNA in situ signal and the GFP RNA in situ signal co-localize in the embryonic brain at the developmental stages NS12 (C) and NS13 (C’, C’’; two different focal planes) indicating that G10011-GFP is a faithful reporter of *TC007335* RNA expression (for additional evidence refer to Figure 6). The asterisk in (C) marks the stomodeum. The expression of *TC007335* RNA in the stomodeum is very low as compared to the expression of GFP-RNA. Scale bars: (B-B") 50 µm; (C-C") 20 µm.

Development • Supplementary information

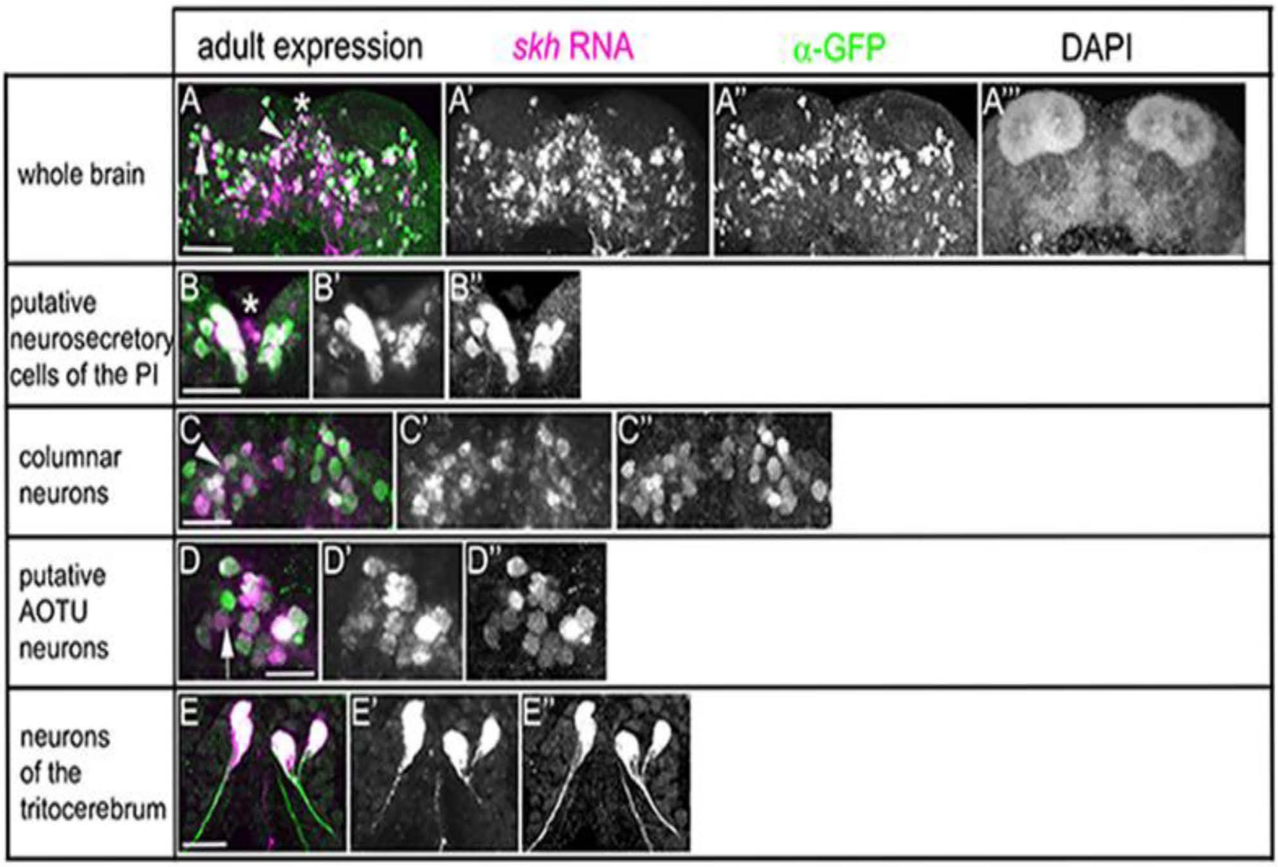

Fig. S5. The *TC007335* RNA in situ signal and the G10011-GFP protein signal largely (and possibly) wholly co-localize in the adult brain. (A) asterix: putative neurosecretory cells of the PI; arrowhead: columnar neurons; arrow: putative AOTU neurons. (A') *skh*-RNA. (A'') G10011-GFP protein. (A''') DAPI staining indicates all nuclei. (B-B'') putative neurosecretory cells of the PI. (B') *skh*-RNA. (B'') G10011-GFP protein. (C-C'') Columnar neurons. (C') *skh*-RNA. (C'') G10011-GFP protein. (D-D'') putative AOTU neurons. (D') *skh*-RNA. (D'') G10011-GFP protein. (E- E`') neurons of the tritocerebrum. (E`) *skh*-RNA. (E`') G10011-GFP protein. The *skh*-RNA and GFP protein signals colocalize in all panels (B-E''). Scale bars: (A-A''') 50 µm; (B-E'') 20 µm.

Development • Supplementary information

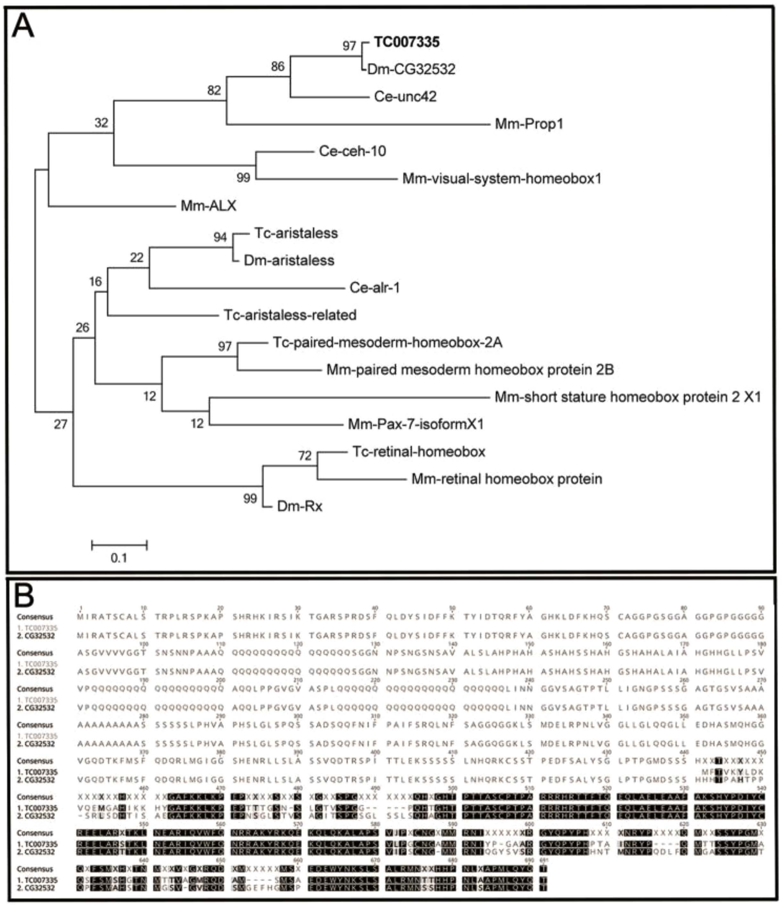

Fig. S6. Phylogenetic tree. (A) Phylogenetic tree reveals *D. melanogaster CG32532*, *C. elegans unc42* and *M. musculus Prop1* as single orthologs of *TC007335*. The TC007335 protein sequence was used to search the NCBI Ref-Seq databases for these species for the most similar proteins using blastp. Alignment was done using the Muscle algorithm as implemented in MEGA 6. The alignment was trimmed to remove all sequences with unclear alignment or gaps. We used Maximum Likelihood, UPGMA and neighbor joining algorithms as implemented in MEGA 6 with bootstrapping based on 500 replications to construct the phylogenetic tree. With all algorithms, the same orthology group is found (shown is the maximum likelihood tree with bootstrap values based on 500 replicates). (B) Protein sequence comparison of *Tc*-Skh and *Dm*-Skh. *Tc*-Skh (229aa) is considerably smaller than the largest predicted *Dm*-Skh isoform (RB-D) (691aa).

Development • Supplementary information

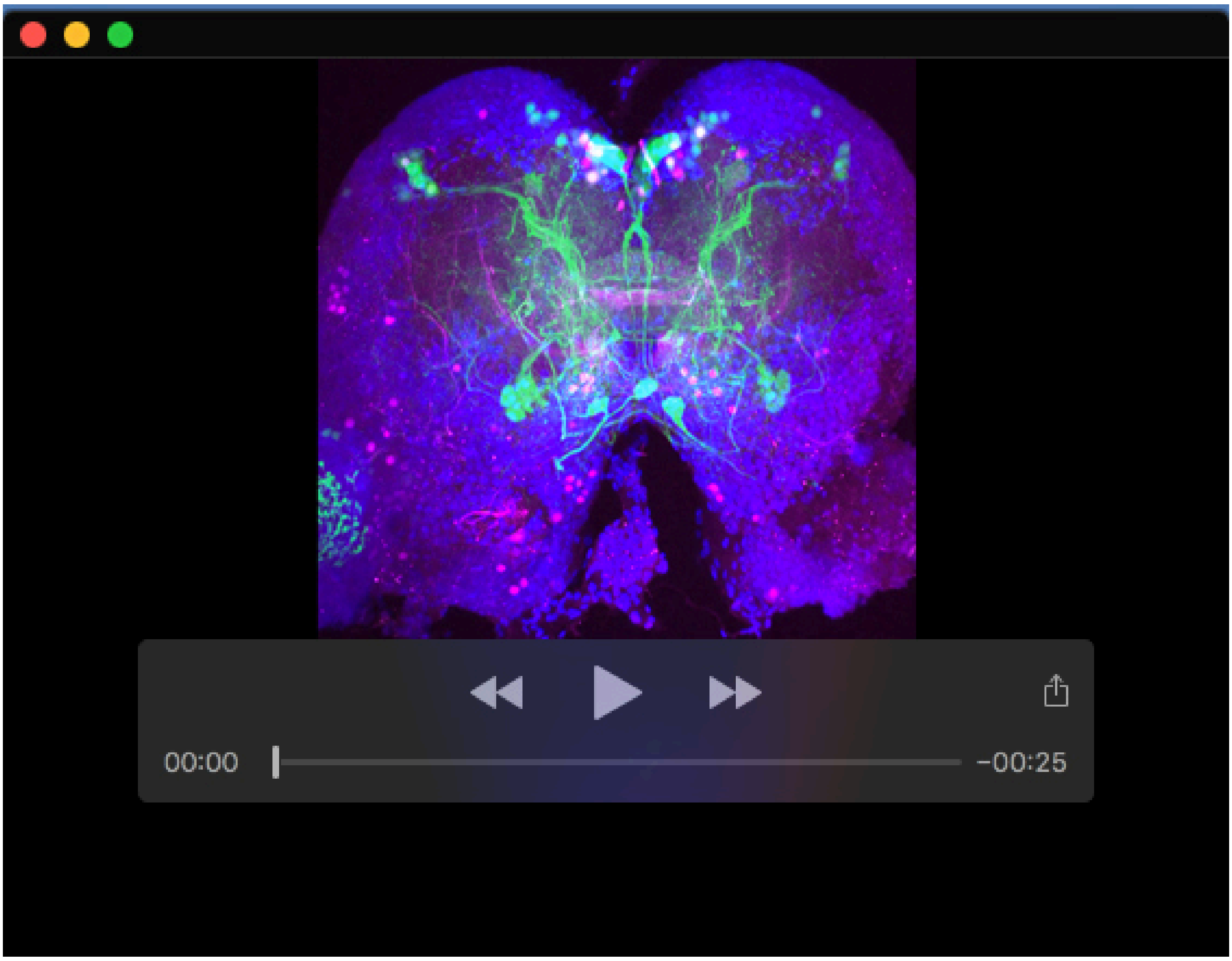

Movie 1. G10011-GFP;5'rx-RFP adult brain

Table S1. Key resources

Reagent type (species) or resource	Designation	Source or reference	Identifiers	Additional information
Genetic reagent (*Tribolium castaneum*)	San Bernadino		SB	Wild type
Genetic reagent (*Tribolium castaneum*)	5'-*rx*-RFP	He et al., 2019		RFP-expression under control of *rx*-upstream region
Genetic reagent (*Tribolium castaneum*)	Ten-a-Δ-RFP	He et al., 2019<br>This study		RFP-expression in MB under the control of *Ten-a*
Genetic reagent (*Tribolium castaneum*)	G10011-GFP	This study		GFP-expression under the control of *skh*
Genetic reagent (*Drosophila melanogaster*)	Oregon R	Bloomington Drosophila Stock Center	RRID:BDSC.5	Wild type
Antibody	anti-GFP (chicken, polyclonal)	Abcam	RRID:AB_300798	IF (1:1000)
Antibody	anti-PH3 (rabbit, polyclonal)	Upstate	RRID:AB_310177	IF (1:100)
Antibody	anti-Repo (rabbit, polyclonal)	von Hilchen et al., 2013		IF (1:1000)
Antibody	anti-Rx (guinea pig, polyclonal)	Farnworth et al., 2020		IF (1:700)
Antibody	anti-Synapsin (mouse, monoclonal)	DSHB	RRID:AB_528479	IF (1:50)
Antibody	anti-acetylated Tubulin (mouse, monoclonal)	Sigma	RRID:AB_609894	IF (1:50)
Antibody, secondary	Goat anti-chicken Alexa Fluor 488	ThermoFisher	RRID:AB_2534096	IF (1:1000)
Antibody, secondary	Goat anti-rabbit Alexa Fluor 555	ThermoFisher	RRID:AB_2535851	IF (1:1000)
Antibody, secondary	Goat anti-mouse Alexa Fluor 555	ThermoFisher	RRID:AB_2535846	IF (1:1000)
Antibody, secondary	Goat anti-guinea pig Alexa Fluor 555	ThermoFisher	RRID:AB_2534117	IF (1:1000)

Development • Supplementary information

Genetic reagents used in each experiment

***Tribolium castaneum* genotypes**	
G10011-GFP;Ten-aΔ-RFP	Fig. 1A-D, Fig. 2L,M
G10011-GFP;5'-*rx*RFP	Fig. 2I-J''
San Bernadino	Fig. S2
G10011-GFP	all other Figs except Fig. 9

***Drosophila melanogaster* line**	
Oregon R	Fig. 9

Sequence-based reagents

Name	Sequence	
TC007335 RNA *in situ* probe	Forward primer:	CTGTGAAGTATTTGGACAAAGTACAAG
	Reverse primer:	GGATGATGCGTTGTGTTCATCCTTAGG
TC007335 dsRNA fragment1	Forward primer:	GCTGAAACCGGAGCCAACGACGACGGGCAG
	Reverse primer:	CTTGCTTTCGGTACTTGGCTCTTCGC
TC007335 dsRNA fragment2	Forward primer:	CTGCAACGGCGCCATGATGCGT
	Reverse primer:	GGATGATGCGTTGTGTTCATCCTTAGG
CG32532 RNA *in situ* probe	Forward primer:	CACCAACAGATGCTCACACGCAGG
	Reverse primer:	GGGAGCCGCTGCAGCAACTATGGC

Development • Supplementary information
