# Supplementary material for: Shaking hands is a homeodomain transcription factor that controls axon outgrowth of central complex neurons in the insect model Tribolium
Source: Development. 2021 Oct 13;148(19):dev199368. doi: 10.1242/dev.199368 (PMC8543150; doi:10.1242/dev.199368)
Supplement: Reviewer comments [file develop-148-199368_review_history.pdf]

The homeodomain transcription factor Shaking hands controls axon outgrowth of central complex neurons in the insect model *Tribolium*

Natalia Carolina Garcia-Perez, Gregor Bucher and Marita Buescher
DOI: 10.1242/dev.199368

Editor: Cassandra Extavour

Review timeline

Original submission:	15 December 2020
Editorial decision:	8 February 2021
First revision received:	3 June 2021
Editorial decision:	5 July 2021
Second revision received:	29 July 2021
Accepted:	9 August 2021

Original submission

First decision letter

MS ID#: DEVELOP/2020/199368

MS TITLE: The homeodomain transcription factor Shaking hands controls axon outgrowth of central complex neurons in the insect model Tribolium

AUTHORS: Natalia Carolina Garcia-Perez, Gregor Bucher, and Marita Buescher

I have now received all the referees reports on the above manuscript, and have reached a decision. The referees' comments are appended below, or you can access them online: please go to BenchPress and click on the 'Manuscripts with Decisions' queue in the Author Area.

The overall evaluation is positive and we would like to publish a revised manuscript in Development, provided that the referees' comments can be satisfactorily addressed. Please attend to all of the reviewers' comments in your revised manuscript and detail them in your point-by-point response. If you do not agree with any of their criticisms or suggestions explain clearly why this is so.

We are aware that you may currently be unable to access the lab to undertake experimental revisions. If it would be helpful, we encourage you to contact us to discuss your revision in greater detail. Please send us a point-by-point response indicating where you are able to address concerns raised (either experimentally or by changes to the text) and where you will not be able to do so within the normal timeframe of a revision. We will then provide further guidance. Please also note that we are happy to extend revision timeframes as necessary.

Reviewer 1

Advance summary and potential significance to field

The authors present two sets of results. In the first (largest) part of the manuscript, they describe the expression pattern of the Tribolium castaneum GFP line G10011. The manuscript convincingly

© 2021. Published by The Company of Biologists under the terms of the Creative Commons Attribution License (https://creativecommons.org/licenses/by/4.0/).

shows that GFP is expressed in the cell cortex and neuropiles of the central complex (CX). The analysis is very detailed covers all developmental stages (embryo to adult) and identifies various neuronal subtypes that contribute to the CX (e.g. ring neurons, anterior optic tubercle neurons, columnar neurons). Furthermore, double-marking with another imaging line reveals that G10011 does not label all cells of the CX substructures (fan-shaped body & ellipsoid body). Lack of co-expression with a glial and a mitotic marker, respectively, suggests that G10010 is expressed in postmitotic neurons.

In the second part, the authors associate the expression of G10011-GFP with the transcription factor TC007335 which they name ‘shaking hands’ (skh). The co-expression shown in Fig. 6 supports this assumption, together with the proximity of the gene to the plasmid insertion site. In the next step, the authors perform RNAi and present an image showing that GFP positive axon fascicles are not present in the protocerebral commissure. They conclude that axon outgrowth is not affected in GFP positive cells but that “axons terminate prematurely close to the respective cell bodies”. Furthermore, the authors state that axon extension is normal in GFP negative cells, based on staining with a general cytoskeletal marker. In addition the authors report that 15% of the GFP positive cells are missing in skh RNAi. Finally, the authors identify a skh ortholog in Drosophila, which has not been characterized yet, and show that it is expressed in a similar pattern as in Tribolium.

Overall, the G10011 is an excellent tool for studying brain development and the authors present a wealth of valuable data. The GFP expression study is meticulous and the conclusions are well supported by the images.
However, there are some issues with analysis and the presentation of the RNAi data and the focus of the discussion.

Comments for the author

Major issues
The authors conclude that the G10011-GFP line “faithfully reports” skh expression based on co-expression studies in the embryo and adult. There certainly is a good amount of overlap but the resolution of the images (Fig. 8) does not allow for evaluating if skh transcripts and GFP protein overlap in all areas. The authors also claim that skh is expressed in the stomodeum, similar to G10011-GFP, but the expression is not visible in the images. Data on other embryonic stages are not shown. The authors should present high resolution images that clearly show that skh transcripts and GFP protein are co-expressed throughout. The images should be properly annotated. Additional stages could be shown in the supplement.

The analysis of the skh RNAi phenotype is superficial. Considering that the authors have put in a lot of work into characterizing the expression of the G10011-GFP line, I would expect a more detailed description of the RNAi phenotype. Are the progeny of DM1-DM4 present? Do they extend axons? Do they fasciculate? Where do they stall? In mild phenotypes, do you see GFP positive axons cross the midline and do they defasiculate?

Do skh RNAi progeny survive into adulthood? Could you show in what ways the CX is affected in the larval and adult skh RNAi brains? Is GFP still expressed in adult CX neurons that derive from embryonic lineages?

Also with regard to RNAi: the authors state “Loss of Tc-Skh has drastic consequences for the outgrowth of all G10011-GFP positive axons: in severely affected embryos, no contra-laterally projecting axons enter the commissural system and hence, no prFB is formed”. Since a (GFP negative) commissure is still seen in skh RNAi, the authors would have to show (1) that the progeny of DM1-4 do not extend axons and (2) that the FB is absent in the adult. This would also prove that the putative DM1-4 progeny clusters the authors identified act as pioneer neurons to establish the FB tracts. When presenting control and RNAi phenotypes, the authors should take care to show the same DV levels (e.g. Fig. 8A and C are clearly not showing the same DV level).

The GFP line description covers ¾ of the results part but is only mentioned in a single paragraph in the discussion. The authors should summarise their main findings and clearly state in what ways the description of the GFP pattern contributes to our knowledge of CX development. In particular, they

© 2021. Published by The Company of Biologists under the terms of the Creative Commons Attribution License (https://creativecommons.org/licenses/by/4.0/).

should discuss how the data compare to the recent PLoS Biology publication by one of the authors (Bucher) on CX development in Tribolium and Drosophila. A thorough comparison to other insects should also be provided.

Instead of discussing at length a potential role of skh as terminal selector gene and the concept of terminal selector genes in C. elegans, the authors should focus on the interpretation of their RNAi results. Additional evidence would be required to confirm a role as terminal selector, as the authors rightly observe.

Minor issues
Title: Should be changed to “The homeodomain transcription factor Shaking hands affects axon outgrowth of central complex neurons in the insect model Tribolium castaneum

Abstract
Line 28: The first sentence of the abstract should be removed – the statement is too general.
Lines 30-33: Phrasing: Three subsequent sentences start with ‘The CX…’
Line 36: This sentence should be removed. The authors have not unambiguously shown that skh is continuously expressed in the same cells throughout development and in adulthood.
Line 37: Add comma: In the embryonic brain, Tc-skh expression

Introduction
Line 46: The authors should say that they describe the developmental mechanisms in Drosophila.
Line 48: Too general and not true in all cases; also contradicts the description of NB and subtype identity further below. “Subtype identity is determined in the early postmitotic neuron and during development it effects proper axon pathfinding thus facilitating the formation of specific neural connections.”
Line 74: Not clear “A role for terminal selectors in the Drosophila brain has not been demonstrated as yet.” Do the authors mean to say that terminal selectors have not been identified yet?
Line 77: Surely there are other areas of the NS that are currently analysed in this context? “Current interest in the specification of subtype identity is focused on neurons whose trajectories build up the central complex (CX) (Boyan and Reichert, 2011; Sullivan et al., 2019; Hartenstein et al., 2020)”
Line 91: Replace slices by subdivisions. “Within the FB, neuronal trajectories are organized to form an intricate substructure of horizontal strata and vertical slices.”
Line 98: References? “The best studied group of small-field neurons are the columnar neurons.”
Line 101: Reference? “Further anterior-ventrally, columnar neurons project four prominent bilateral pairs of fiber bundles (w, x, y and z tracts).”
Line 123: Misleading. Add ….at different stages of development in different species….“Moreover, the assembly of individual CX neuropils occurs at different stages of development, a phenomenon referred to as heterochrony (Panov, 1959).
Line 125: So, what did you find out? Is this the starting point for this study? “We study the regulatory mechanisms that underlie CX development in the red flour beetle Tribolium castaneum (He et al., 2019; Farnworth et al., 2020).”

Results
Line 151: Data? Reference? “G10011 beetles are homozygous viable, fertile and their lifespan is comparable to that of the Tribolium wild type strain SB.”
Line 155: Putting G10011 expression into context, rather than gaining an overview. “To gain an overview of G10011-GFP expression, we crossed G10011 to Ten-a-Δ-RFP expressing beetles and…”
Line 161 (Fig. 1 legend): There seems to be expression in the OL and the VNC: “Note that Ten-a-Δ-RFP expression is restricted to the mushroom bodies (MB; magenta)”
Line 169: (Fig. 1B): What do the arrows point to? There is only one arrowhead.
Line 175: (Fig. 1C): Label the approximate borders of PC, DC, TC
Line 199: Why is “hugin expressing” shown in quotation marks?
Line 235: Abbreviation not explained: BU
Line 259: Typo: posteriorly “…….one of which extends first medially and then posterior before it enters the uppermost stratum of the FB…..”
Line 274: Typos: “(Embryonic are given oordinations according to the body axis)”
Line 285: Error: arrowhead. “(C) Note that multiple GFP-positive cell clusters project their axons from posterior dorsomedial regions towards the primary brain commissure (arrow).”

© 2021. Published by The Company of Biologists under the terms of the Creative Commons Attribution License (https://creativecommons.org/licenses/by/4.0/).

Line 295: Typo: The. "(E') the four clusters of neurons produce four parallel running GFP-positive…."
Line 301: Not visible. "Note the beginning defasciculation of GFP-positive commissural fiber tracts (white lines)."
Line 319: Reference? "The stem cells of the brain generate their neural progeny in a stereotyped orientation towards the inside of the brain."
Line 320: Order of figures: Either show this image in Fig. 2 or refer to this point, when discussing results of Fig. 6. "Sequentially generated neurons remain close together such that lineage-related neurons appear like pearls on a string. GFP RNA in situ in stage NS15 G10011 embryos allows us to identify 14-16 "strings" of GFP323 positive cells (Fig. 6B'')."
Line 330: Without e.g. lineage tracing, you cannot be sure that the same cells express GFP throughout development and into adulthood. "We asked whether these cells are of embryonic origin and establish the FB primordium (prFB) of the embryonic Tribolium brain."
Line 332: Misleading. ….produced by the progeny of four distinct neuroblasts…"These fiber tracts are produced by four distinct neuronal neuroblasts (DM1-DM4) located in the posterior-medial brain (Andrade et al., 2019; Farnworth et al., 2020).
Line 340: Replace 'fuse' by 'fasciculate'. "……..four contralaterally projecting fiber tracts enter the commissural system as parallel tracts and fuse with the corresponding tracts of the opposing brain hemisphere." This is not seen on the image. How do you know that only the corresponding tracts fasciculate?
Line 342: Reference? "At late stage NS15 these fibers show the characteristic pattern of defasciculation which initiates the development of the columnar architecture of the FB"
Line 359: Typo (hugin). "….. as well as the putative "huging" expressing cells (arrowhead) and the neurosecretory cells of the prospective PI….."
Line 361: Terminology. The longitudinal nerve tracts that run from the brain through the VNC are called longitudinal connectives. Replace GFP-positive longitudinal connectives by fascicles/axons. "The adult VNC contains numerous GFP-positive longitudinal connectives which originate in the brain"
Line 401: On average or exactly? "In the late larval brain each hemisphere contains 384 GFP-positive cells (n=4)."
Line 404: This sentence is out of place here. TC007335 has not been introduced at this stage. Move to discussion. "Since these tissues are not easily accessible by RNA in situ hybridization, we currently do not know whether fluorescence reflects the bona fide expression of TC007335 (see below) or is due to cryptic regulatory elements within our plasmid."
Line 414: Fig. 5 legend. DAPI not visible except for faint background. "GFP autofluorescence (in A combined with combined with DAPI staining, blue)."
Line 433: Fig. 6. It would be more consistent to use the same colour for GFP and skh, respectively, in all panels.
Line 434: Skh expression is not visible in the stomodeum. "Note that the expression of GFP and skh are restricted to the brain and stomodeum (asterisk).
Line 444; Typo. "skh encodes the ortholog of the C.elegans transcription factor 444 UNC-42"
Line 446: This is not proof of continued expression. The GFP/skh expressing cells might not be the same throughout development. "To investigate whether this reflects GFP perdurance or the continued expression of Tc-skh, we performed skh whole mount RNA in situ combined with Δ-GFP staining in the adult brain. All GFP-positive cells are also skh RNA positive, demonstrating the continued expression of skh (Fig. 6C-C'')."
Line 456: Add 'magenta'. "Embryonic G10011 brains were stained with Δ-GFP (green) and Δ-PH3 (A-A'')."
Line 461: You have not shown that this is the case throughout development. Change to 'We did not observe co-localization of Δ-GFP and Δ-PH-3 signals in the analysed stages…..' "Co-localization of Δ-GFP and Δ-PH-3 signals is never observed, indicating that Tc-skh expression is restricted to postmitotic cells"
Line 464: Amend to 'We conclude that Tc-skh expression is restricted to neurons in adults.' "To examine whether Tc-skh is expressed in glia, we double-stained embryos and adult brains with Δ-GFP and the glial marker Δ-Repo. Δ-GFP and Δ-Repo signals do not co-localize (Fig. 7B-C'' and data not shown). We conclude that Tc-skh expression is restricted to neurons."
Line 483: Fig. 8 legend. Typo "(C) whole brain at low magnification;…"
Line 487: This information should be given in Materials and Methods and in the main text. "commissural defects were scored at stages NS14 and NS15. Buffer-injected control (co) n=80 (2 biological replicates) 3% defects, dsRNA frag1 n=85 (2 biological replicates) 71% defects, dsRNA

© 2021. Published by The Company of Biologists under the terms of the Creative Commons Attribution License (https://creativecommons.org/licenses/by/4.0/).

frag2 n=35, 48% defects. (F) Loss of GFP-positive cells in skh RNAi embryos were scored at NS15. 25 Buffer-injected control (co): 362 GFP-positive cells (n=4), dsRNA frag1: 490 308 GFP-positive cells (n=4). Statistical significance was determined by one way ANOVA, ** = P<0.05."
What types of "defects" were scored? Does the control show commissural defects?
Line 493: Typo, contralaterally "…..in severely affected embryos, no contra-laterally projecting axons ….
Line 497 & 498 : Wrong figure references. "Examination of GFP-fluorescent cells shows that some 497 axon outgrowth still takes place but axons terminate prematurely close to the respective cell 498 bodies (Fig. 7C)." "Axon extension defects are restricted to GFP-positive trajectories: acTubulin positive but GFP negative axon trajectories form normally (Fig. 7C,D'')."
Line 499: Not shown. There are alternative explanations: e.g. the ability to respond to paracrine factors might be affected in skh RNAi. "We conclude that the requirement of Tc-Skh for axon extension is cell-autonomous."
Line 505: Amend to …… whether loss of GFP is due to apoptosis or reflects….. "Due to the lack of specific markers for G10011-GFP positive cells, we are unable to determine whether loss of Skh is due to apoptosis or reflects an auto-regulatory feedback loop in the maintenance of Skh expression.
Line 556: Reference? "Terminal selector expression in neurons is continuous from cell birth to cell death"
Line 567: Not in all cases. Reference? "Maintenance of terminal selector expression is accomplished by positive auto-regulatory feed-back loops; accordingly, loss of terminal selector activity results in the loss of terminal selector gene expression at later stages." In this context an analysis of the RNAi progeny into adulthood would be interesting.
Line 580: This has not been shown. The corresponding figure merely mentions GFP positive clusters. Columnar neurons are not indicated. "Knock-down of Tc-Skh abolishes columnar (and other) neuron axon extensions indicating that a requirement of Skh for the development of proper connectivity is conserved between Tribolium and C. elegans."
Lines 599-606: This paragraph should be removed since it is not related to the analysis. "The activation of terminal selector gene expression is the final step of hierarchical gene regulatory cascades which provide early……"

Reviewer 2

Advance summary and potential significance to field

This manuscript reports the expression of a new homeobox gene, skh, in Tribolium and Drosophila. The authors provide a detailed, very lucid description of how expression relates to different elements of the central complex (CX), and they pay attention to different developmental stages from embryo to adult. The describe the expression of the Drosophila homolog in the embryo, and conduct knockdown studies in Tribolium which yield an interesting, interpretable phenotype. The paper makes a significant contribution to our understanding of brain development in Tribolium, and insects in general.

Comments for the author

Comments:
Line 107-111: are these Tribolium neurons? Reference?
Line 193, 200: references for these neurosecretory cells in Tribolium? In the absence of the relevant markers for the peptides, it may be premature to identify the cells with groups of similar cells described in Drosophila Line 235ff: compartments have been described in dung beetle (Immonen et al 2016, JCN); the authors could refer to this paper.
Fig.2H: what is the strong label ventral of the MB medial lobes?
Line 247ff: when discussing the labeled neurons in more detail:
1.Based on Drosophila (and to some extent dung beetle, which should be incorporated in more detail by the authors) the columnar neurons fall into the groups horizontal system, vertical system, and pontine system (Hanesch et al 1989). Vertical: PB-FB or EB-NO; Horizontal: PB-FB or EB-Gall or Rubus. These large grouping could be used here: the fact that there is little label in noduli implies that the G10011 columnar neurons should not belong to the vertical system; to confirm: is there label in the gall/rubus (dorsal part of LAL)? Rx-positive cells described by authors in a previous publication do have projections to gall.

© 2021. Published by The Company of Biologists under the terms of the Creative Commons Attribution License (https://creativecommons.org/licenses/by/4.0/).

2.Ring neurons: is that term used in beetles? The corresponding neurons have been described for dung beetle, I believe. Given that the beetle EB is not ring-shaped, the axonal projection of “ring” neurons is also not circular. The problem of nomenclature should be discussed.
3.AOTU neurons: I am not clear on this group. Do the neurons have dendritic arbors in the AOTU, or are just cell bodies located close to it? Were neurons with the observed pattern described before, for beetles or Drosophila?
Line 328ff: the paper Boyan et al. 2017 also describes comparatively embryonic FBpr development
Line 401: is the EB detectable with other markers in late larvae, like synapsin or acTub?
Fig.6: I am basically convinced of the authors’ statement of coincidence of skh and G10011. What would help in panel A is to outline (hatched line…) the brain hemispheres to demonstrate that skh, and G10011, are both expressed in a distinct small region of the overall brain.
What explains the different “textures” of the signals in panels B, B’, B’’? the skh RNA is more concentrated in the center of the cell group (these are all somata, right?), the GFP RNA in the periphery. Could the authors comment on that?

Reviewer 3

Advance summary and potential significance to field

The manuscript by Garcia-Perez et al describes the identification and functional characterization of the Shaking hands gene in Tribolium castaneum. This study is very carefully executed, nicely documented and reveals a role of the Shaking hands gene-encoded protein as a terminal neuronal differentiation factor in a subset of neurons that form the central complex. This study makes elegant use of the genetic tools available in Tribolium to start to decipher the genetic control of central complex development thereby also opening the possibility to start a systematic comparative analysis with Drosophila melanogaster where central complex development has been studied most extensively.

Comments for the author

1. On lines 74-75, the authors state that a role for terminal selectors has not been demonstrated yet in Drosophila. I do not think this is correct (e.g. the work of Stefan Thor and others). Please verify and adjust this statement as needed.

2. On page 3-5, the authors give a very lengthy description of the central complex in Drosophila. I understand why this was done, given the extensive description of Drosophila CX anatomy. However, it would be very useful to also specify the (dis)similarities of the T. castaneum CX versus Drosophila. One example is the shape of the ellipsoid body.

3. In line with the previous comment, the authors state on lines 112-113 that the architecture of the adult CX is well documented in many insect species. However in a later statement they only refer to Drosophila and locust work (lines 247-251). Can the authors clarify this? When saying many insect species, how many?

Different insect orders?
4. On line 188 (and elsewhere in the manuscript) the authors refer to n-dorsal or n-ventral brain. What does the n stand for?

5. On lines 192-200, the authors argue that based on cell body location (and I assume size) that the neurons in the pars intercerebralis are neurosecretory and those in the tritocerebrum are likely to be hugin-expressing cells.
In the absence of positive labeling for neurosecretory cells and even more so for hugin, please provide more rationale. Is hugin encoded in the Tribolium genome? It would be more convincing to do a double-labeling experiment (e.g. immuno and in situ).

6. On line 255, the authors state: 'four characteristic fiber tracts commonly named ...'
Why commonly?

© 2021. Published by The Company of Biologists under the terms of the Creative Commons Attribution License (https://creativecommons.org/licenses/by/4.0/).

7. On line 274, the sentence is a bit strange. 'Embryonic are given oordinations according to the body axis'. I am not sure the word oordination exists. Is meant here, 'Embryonic stages are given coordinates according to the body axis'? Please clarify.

8. In the legend of figure 3, there are a few typos and one term that I am not sure of.
The authors refer to 'stomodeum'. Is that the correct name at this developmental stage? Or should it be foregut?

Typos:
line 296: GFP-positive fascicles THAT enter the commissural ...
line 303: Multiple GFP-positive fibers exist IN the brain ...
9. In figure 3, please consider replacing the dark blue color in panel K by white or grey. I am not sure it will reproduce well. At least I was not able to distinguish the label very well.

10. Line 312: small clusters OF GFP-positive ...

11. Lines 323-327: How do you come to the conclusion that G10011-GFP is expressed in the progeny of about 15% of all NB lineages?

12. Line 359: putative HUGIN expressing cells

13. Line 441: FAITHFULLY

14. Line 445: It would be nice to include in Fig. S6 a protein sequence alignment in particular of the Tribolium and Drosophila proteins.

15. Lines 464-467: The antibody against Repo that was used to label glia. Since this is generated against the Drosophila Repo protein, how was this validated for use in Tribolium? Does it recognize a protein of the correct size in Tribolium (tested on Western blot)?

16. Lines 494-496. Do the axon outgrowth defects correlate with knockdown efficiency? Was this quantified?

17. Why was the analysis of Dm skh limited to the embryo? Would in situ analysis on larval (e.g. L3) and adult brain not provide further corroborating evidence? In particular given the link with the central complex, parts of which develop only later in development in Drosophila.

First revision

<u>Author response to reviewers' comments</u>

Reviewer 1 Advance summary and potential significance to field
The authors present two sets of results. In the first (largest) part of the manuscript, they describe the expression pattern of the Tribolium castaneum GFP line G10011. The manuscript convincingly shows that GFP is expressed in the cell cortex and neuropiles of the central complex (CX). The analysis is very detailed, covers all developmental stages (embryo to adult) and identifies various neuronal subtypes that contribute to the CX (e.g. ring neurons, anterior optic tubercle neurons, columnar neurons). Furthermore, double-marking with another imaging line reveals that G10011 does not label all cells of the CX substructures (fan-shaped body & ellipsoid body). Lack of co-expression with a glial and a mitotic marker, respectively, suggests that G10010 is expressed in postmitotic neurons.

In the second part, the authors associate the expression of G10011-GFP with the transcription factor TC007335 which they name ‘shaking hands’ (skh). The co-expression shown in Fig. 6 supports this assumption, together with the proximity of the gene to the plasmid insertion site. In the next step, the authors perform RNAi and present an image showing that GFP positive axon fascicles are

© 2021. Published by The Company of Biologists under the terms of the Creative Commons Attribution License (https://creativecommons.org/licenses/by/4.0/).

not present in the protocerebral commissure. They conclude that axon outgrowth is not affected in GFP positive cells but that “axons terminate prematurely close to the respective cell bodies”. Furthermore, the authors state that axon extension is normal in GFP negative cells, based on staining with a general cytoskeletal marker. In addition, the authors report that 15% of the GFP positive cells are missing in skh RNAi. Finally, the authors identify a skh ortholog in Drosophila, which has not been characterized yet, and show that it is expressed in a similar pattern as in Tribolium.

Overall, the G10011 is an excellent tool for studying brain development and the authors present a wealth of valuable data. The GFP expression study is meticulous and the conclusions are well supported by the images.
However, there are some issues with analysis and the presentation of the RNAi data and the focus of the discussion.

Reviewer 1 Comments for the author

Major issues

The authors conclude that the G10011-GFP line “faithfully reports” skh expression based on co-expression studies in the embryo and adult. There certainly is a good amount of overlap but the resolution of the images (Fig. 8) does not allow for evaluating if skh transcripts and GFP protein overlap in all areas. The authors also claim that skh is expressed in the stomodeum, similar to G10011-GFP, but the expression is not visible in the images. Data on other embryonic stages are not shown. The authors should present high resolution images that clearly show that skh transcripts and GFP protein are co-expressed throughout. The images should be properly annotated. Additional stages could be shown in the supplement.
We now show data on additional embryonic stages in Figure S5 C-C’’. The reviewer is correct when stating that skh RNA expression in the stomodeum is not visible in Figure 8. For reasons unknown to us, skh RNA expression in the stomodeum is significantly weaker as the GFP RNA expression. Nevertheless, expression of skh in the stomodeum is there and we show this now in Figure S5 C. We would like to point out that the reviewer has misunderstood the experiment depicted in Figure 8: we don’t show the combination of skh RNA and GFP protein but the combination of skh and GFP RNAs.

The analysis of the skh RNAi phenotype is superficial. Considering that the authors have put in a lot of work into characterizing the expression of the G10011-GFP line, I would expect a more detailed description of the RNAi phenotype. Are the progeny of DM1-DM4 present? *Yes*. Do they extend axons? *Yes* Do they fasciculate? *Yes* Where do they stall?
We have added a sentence referring specifically to the progeny of DM1-DM4.
In severe phenotypes close to the cell bodies. In mild phenotypes, do you see GFP positive axons cross the midline? In mild phenotypes some GFP positive axons enter the commissural system; and do they defasiculate? *We don’t knowskh RNAi animals die at late embryogenesis and/or shortly after larval hatching precluding the experiments suggested by the reviewer*. Do skh RNAi progeny survive into adulthood? Could you show in what ways the CX is affected in the larval and adult skh RNAi brains? Is GFP still expressed in adult CX neurons that derive from embryonic lineages?

Also with regard to RNAi: the authors state “Loss of Tc-Skh has drastic consequences for the outgrowth of all G10011-GFP positive axons: in severely affected embryos, no contra-laterally projecting axons enter the commissural system and hence, no prFB is formed”. Since a (GFP negative) commissure is still seen in skh RNAi, the authors would have to show (1) that the progeny of DM1-4 do not extend axons and (2) that the FB is absent in the adult. This would also prove that the putative DM1-4 progeny clusters the authors identified act as pioneer neurons to establish the FB tracts. RNAi animals do not survive to adulthood.
When presenting control and RNAi phenotypes, the authors should take care to show the same DV levels (e.g. Fig. 8A and C are clearly not showing the same DV level).
Panels 8A and C are both focused to show the fibers that enter the commissural system. Since no two brains are ever mounted in a perfectly equal manner a slight shift in the DV levels is common. To give all readers the opportunity to view the original unprocessed image stacks we have uploaded them to figshare.

© 2021. Published by The Company of Biologists under the terms of the Creative Commons Attribution License (https://creativecommons.org/licenses/by/4.0/).

The GFP line description covers ¾ of the results part but is only mentioned in a single paragraph in the discussion. The authors should summarise their main findings and clearly state in what ways the description of the GFP pattern contributes to our knowledge of CX development. In particular, they should discuss how the data compare to the recent PLoS Biology publication by one of the authors (Bucher) on CX development in Tribolium and Drosophila. A thorough comparison to other insects should also be provided.
We have extended our discussion by a short comparison to other insects. A thorough comparison is beyond the scope of this manuscript.

Instead of discussing at length a potential role of skh as terminal selector gene and the concept of terminal selector genes in C. elegans, the authors should focus on the interpretation of their RNAi results. Additional evidence would be required to confirm a role as terminal selector, as the authors rightly observe.
The discussion of skh as a terminal selector has been significantly shortened.

Minor issues

Title: Should be changed to “The homeodomain transcription factor Shaking hands affects axon outgrowth of central complex neurons in the insect model Tribolium castaneum
The title has been changed

Abstract

Line 28: The first sentence of the abstract should be removed – the statement is too general.
removed

Lines 30-33: Phrasing: Three subsequent sentences start with ‘The CX…
rephrased

Line 36: This sentence should be removed. The authors have not unambiguously shown that skh is continuously expressed in the same cells throughout development and in adulthood.
rephrased

Line 37: Add comma: In the embryonic brain, Tc-skh expression

done

Introduction

Line 46: The authors should say that they describe the developmental mechanisms in Drosophila.
This is already written in the text: lines 48-49.

Line 48: Too general and not true in all cases; also contradicts the description of NB and subtype identity further below. “Subtype identity is determined in the early postmitotic neuron and during development it effects proper axon pathfinding thus facilitating the formation of specific neural connections.”
We agree with the reviewer that the term “determined” is misleading in this context. We have rephrased the statement.

Line 74: Not clear “A role for terminal selectors in the Drosophila brain has not been demonstrated as yet.” Do the authors mean to say that terminal selectors have not been identified yet?
Terminal selectors have been studied in the ventral nerve cord by Thor and others. In our manuscript, we state that no role for terminal selectors has been demonstrated as yet in the Drosophila brain. We think this statement is correct. Nevertheless, we have removed this sentence

Line 77: Surely there are other areas of the NS that are currently analysed in this context? “Current interest in the specification of subtype identity is focused on neurons whose trajectories build up the central complex (CX) (Boyan and Reichert, 2011; Sullivan et al., 2019; Hartenstein et al., 2020)”

© 2021. Published by The Company of Biologists under the terms of the Creative Commons Attribution License (https://creativecommons.org/licenses/by/4.0/).

rephrased

Line 91: Replace slices by subdivisions. “Within the FB, neuronal trajectories are organized to form an intricate substructure of horizontal strata and vertical slices.”

done

Line 98: References? “The best studied group of small-field neurons are the columnar neurons.”

rephrased

Line 101: Reference? “Further anterior-ventrally, columnar neurons project four prominent bilateral pairs of fiber bundles (w, x, y and z tracts).”

A reference has been added

Line 123: Misleading. Add ….at different stages of development in different species….“Moreover, the assembly of individual CX neuropils occurs at different stages of development, a phenomenon referred to as heterochrony (Panov, 1959).

This part has been removed.

Line 125: So, what did you find out? Is this the starting point for this study? “We study the regulatory mechanisms that underlie CX development in the red flour beetle Tribolium castaneum (He et al., 2019; Farnworth et al., 2020).”

Well, we identified a transcription factor that is required for axon outgrowth of a subset of CX neurons. This is stated several times in the manuscript.

Results

Line 151: Data? Reference? “G10011 beetles are homozygous viable, fertile and their lifespan is comparable to that of the Tribolium wild type strain SB.”

We have now added the data source.

Line 155: Putting G10011 expression into context, rather than gaining an overview. “To gain an overview of G10011-GFP expression, we crossed G10011 to Ten-a-Δ-RFP expressing beetles and…”

done

Line 161 (Fig. 1 legend): There seems to be expression in the OL and the VNC: “Note that Ten-a-Δ-RFP expression is restricted to the mushroom bodies (MB; magenta)”

rephrased

Line 169: (Fig. 1B): What do the arrows point to? There is only one arrowhead.

Amended

Line 175: (Fig. 1C): Label the approximate borders of PC, DC, TC

done

Line 199: Why is “hugin expressing” shown in quotation marks?

hugin has been removed

Line 235: Abbreviation not explained: BU

© 2021. Published by The Company of Biologists under the terms of the Creative Commons Attribution License (https://creativecommons.org/licenses/by/4.0/).

corrected

Line 259: Typo: posteriorly "…….one of which extends first medially and then posterior before it enters the
uppermost stratum of the FB….."

corrected

Line 274: Typos: "(Embryonic are given oordinations according to the body axis)"

corrected

Line 285: Error: arrowhead. "(C) Note that multiple GFP-positive cell clusters project their axons from posterior dorsomedial regions towards the primary brain commissure (arrow)."

changed

Line 295: Typo: The. "(E') the four clusters of neurons produce four parallel running GFP-positive…."

corrected

Line 301: Not visible. "Note the beginning defasciculation of GFP-positive commissural fiber tracts (white lines)."

We think it is visible in high-resolution images. In addition, defasciculation is also shown in Figure S4 C''.

Line 319: Reference? "The stem cells of the brain generate their neural progeny in a stereotyped orientation towards the inside of the brain."

This part has been removed.

Line 320: Order of figures: Either show this image in Fig. 2 or refer to this point, when discussing results of Fig. 6. "Sequentially generated neurons remain close together such that lineage-related neurons appear like pearls on a string. GFP RNA in situ in stage NS15 G10011 embryos allows us to identify 14-16 "strings" of GFP323 positive cells (Fig. 6B'')."

This part has been removed.

Line 330: Without e.g. lineage tracing, you cannot be sure that the same cells express GFP throughout development and into adulthood. "We asked whether these cells are of embryonic origin and establish the FB primordium (prFB) of the embryonic Tribolium brain."

We think, we that we provide sufficient evidence to support our statement that the embryonic progeny of DM1-DM4 expresses GFP and maintains GFP expression at larval, pupal and adult stages.

Line 332: Misleading. ….produced by the progeny of four distinct neuroblasts…"These fiber tracts are produced by four distinct neuronal neuroblasts (DM1-DM4) located in the posterior-medial brain (Andrade et al., 2019; Farnworth et al., 2020).

changed

Line 340: Replace 'fuse' by 'fasciculate'. "……..four contralaterally projecting fiber tracts enter the commissural system as parallel tracts and fuse with the corresponding tracts of the opposing brain hemisphere." This is not seen on the image. How do you know that only the corresponding tracts fasciculate?

© 2021. Published by The Company of Biologists under the terms of the Creative Commons Attribution License (https://creativecommons.org/licenses/by/4.0/).

We agree with the reviewer that our statement is not supported by the image we provide. We have removed our statement.

Line 342: Reference? “At late stage NS15 these fibers show the characteristic pattern of defasciculation which initiates the development of the columnar architecture of the FB”

We have added the reference.

Line 359: Typo (hugin). “….. as well as the putative “huging” expressing cells (arrowhead) and the neurosecretory cells of the prospective PI…..”

Hugin has been removed

Line 361: Terminology. The longitudinal nerve tracts that run from the brain through the VNC are called longitudinal connectives. Replace GFP-positive longitudinal connectives by fascicles/axons. “The adult VNC contains numerous GFP-positive longitudinal connectives which originate in the brain”

changed

Line 401: On average or exactly? “In the late larval brain each hemisphere contains 384 GFP-positive cells (n=4).”

changed to “on average”

Line 404: This sentence is out of place here. TC007335 has not been introduced at this stage. Move to discussion. “Since these tissues are not easily accessible by RNA in situ hybridization, we currently do not know whether fluorescence reflects the bona fide expression of TC007335 (see below) or is due to cryptic regulatory elements within our plasmid.”

This part has been removed.

Line 414: Fig. 5 legend. DAPI not visible except for faint background. “GFP autofluorescence (in A combined with combined with DAPI staining, blue).”

We have increased the brightness of the blue DAPI staining.

Line 433: Fig. 6. It would be more consistent to use the same colour for GFP and skh, respectively, in all panels.

We use consistently the colour green for all anti-GFP stainings and autofluorescence. We use consistently the colour red for GFP RNA in situs. The different use of colour should make the reader aware of the difference between GFP protein and GFP RNA. See above our response to the first major comment.

Line 434: Skh expression is not visible in the stomodeum. “Note that the expression of GFP and skh are restricted to the brain and stomodeum (asterisk).

We have added additional panels to Figure S 5 (C) to demonstrate skh RNA expression in the stomodeum.

Line 444; Typo. “skh encodes the ortholog of the C.elegans transcription factor 444 UNC-42”
corrected

Line 446: This is not proof of continued expression. The GFP/skh expressing cells might not be the same throughout development. “To investigate whether this reflects GFP perdurance or the continued expression of Tc-skh, we performed skh whole mount RNA in situ combined with Δ-GFP staining in the adult brain. All GFP-positive cells are also skh RNA positive, demonstrating the continued expression of skh (Fig. 6C-C’’).”

© 2021. Published by The Company of Biologists under the terms of the Creative Commons Attribution License (https://creativecommons.org/licenses/by/4.0/).

We believe that we provide sufficient evidence at least for some neurons that can be identified in the embryonic brain and coniously observed throughout development: the columnar neurons, the putative neurosecretory neurons of the pars intercerebralis.

Line 456: Add 'magenta'. "Embryonic G10011 brains were stained with Δ-GFP (green) and Δ-PH3 (A-A'')."

done

Line 461: You have not shown that this is the case throughout development. Change to 'We did not observe co-localization of Δ-GFP and Δ-PH-3 signals in the analysed stages…..' "Co-localization of Δ-GFP and Δ-PH-3 signals is never observed, indicating that Tc-skh expression is restricted to postmitotic cells"

we have changed this accordingly.

Line 464: Amend to 'We conclude that Tc-skh expression is restricted to neurons in adults.' "To examine whether Tc-skh is expressed in glia, we double-stained embryos and adult brains with Δ-GFP and the glial marker Δ-Repo. Δ-GFP and Δ-Repo signals do not co-localize (Fig. 7B-C'' and data not shown). We conclude that Tc-skh expression is restricted to neurons."

amended

Line 483: Fig. 8 legend. Typo "(C) whole brain at low magnification;…"

corrected

Line 487: This information should be given in Materials and Methods and in the main text. "commissural defects were scored at stages NS14 and NS15. Buffer-injected control (co) n=80 (2 biological replicates) 3% defects, dsRNA frag1 n=85 (2 biological replicates) 71% defects, dsRNA frag2 n=35, 48% defects. (F) Loss of GFP-positive cells in skh RNAi embryos were scored at NS15. 25 Buffer-injected control (co): 362 GFP-positive cells (n=4), dsRNA frag1: 490 308 GFP-positive cells (n=4). Statistical significance was determined by one way ANOVA, ** = P<0.05."
What types of "defects" were scored? Does the control show commissural defects?

We maintain that the best place for this information is the figure legend.
We scored the partial and/or complete loss of GFP-positive commissural fibers in at least one hemisphere. The control shows similar defects with a very low frequency (3%).

Line 493: Typo, contralaterally "…..in severely affected embryos, no contra-laterally projecting axons ….

corrected

Line 497 & 498 : Wrong figure references. "Examination of GFP-fluorescent cells shows that some 497 axon outgrowth still takes place but axons terminate prematurely close to the respective cell 498 bodies (Fig. 7C)." "Axon extension defects are restricted to GFP-positive trajectories: acTubulin positive but GFP negative axon trajectories form normally (Fig. 7C,D'')."

amended

Line 499: Not shown. There are alternative explanations: e.g. the ability to respond to paracrine factors might be affected in skh RNAi. "We conclude that the requirement of Tc-Skh for axon extension is cell-autonomous."

rephrased

Line 505: Amend to …… whether loss of GFP is due to apoptosis or reflects….. "Due to the lack of specific markers for G10011-GFP positive cells, we are unable to determine whether loss of Skh is due to apoptosis or reflects an auto-regulatory feedback loop in the maintenance of Skh expression.

© 2021. Published by The Company of Biologists under the terms of the Creative Commons Attribution License (https://creativecommons.org/licenses/by/4.0/).

amended

Line 556: Reference? "Terminal selector expression in neurons is continuous from cell birth to cell death"

reference added

Line 567: Not in all cases. Reference? "Maintenance of terminal selector expression is accomplished by positive auto-regulatory feed-back loops; accordingly, loss of terminal selector activity results in the loss of terminal selector gene expression at later stages." In this context an analysis of the RNAi progeny into adulthood would be interesting.

Indeed, an analysis of postembryonic skh RNAi progeny would be very interesting. However, as stated above, skh RNAi (at 2ug/ul) is largely embryonic lethal with few larval escapers.

Line 580: This has not been shown. The corresponding figure merely mentions GFP positive clusters. Columnar neurons are not indicated. "Knock-down of Tc-Skh abolishes columnar (and other) neuron axon extensions indicating that a requirement of Skh for the development of proper connectivity is conserved between Tribolium and C. elegans."

rephrased.
Lines 599-606: This paragraph should be removed since it is not related to the analysis. "The activation of terminal selector gene expression is the final step of hierarchical gene regulatory cascades which provide early……"

removed

Reviewer 2 Advance summary and potential significance to field
This manuscript reports the expression of a new homeobox gene, skh, in Tribolium and Drosophila. The authors provide a detailed, very lucid description of how expression relates to different elements of the central complex (CX), and they pay attention to different developmental stages from embryo to adult. The describe the expression of the Drosophila homolog in the embryo, and conduct knockdown studies in Tribolium which yield an interesting, interpretable phenotype. The paper makes a significant contribution to our understanding of brain development in Tribolium, and insects in general.

Reviewer 2 Comments for the author

Comments:
Line 107-111: are these Tribolium neurons? Reference?

no, they are Drosophila neurons

Line 193, 200: references for these neurosecretory cells in Tribolium? In the absence of the relevant markers for the peptides, it may be premature to identify the cells with groups of similar cells described in Drosophila
The reviewers are correct to state that we have not proven the neurosecretory character of these cells. However, these Tribolium neurons share more than their position, morphology and axon projections with the corresponding Drosophila cells: they have the same NB origin and express the same molecular markers. These data are not included in our manuscript as we feel it would take the focus of the CX. In our manuscript, we describe these cells putative neurosecretory. We feel this statement is justified.

Line 235ff: compartments have been described in dung beetle (Immonen et al 2016, JCN); the authors could refer to this paper.
Thank you for pointing out the dung beetle study to us. It had escaped our attention. We now compare our anatomical findings with those of the dung beetle in the discussion.

Fig.2H: what is the strong label ventral of the MB medial lobes?
We don't know.

© 2021. Published by The Company of Biologists under the terms of the Creative Commons Attribution License (https://creativecommons.org/licenses/by/4.0/).

Line 247ff: when discussing the labeled neurons in more detail:
1.Based on Drosophila (and to some extent dung beetle, which should be incorporated in more detail by the authors) the columnar neurons fall into the groups horizontal system, vertical system, and pontine system (Hanesch et al 1989). Vertical: PB-FB or EB-NO; Horizontal: PB-FB or EB-Gall or Rubus. These large grouping could be used here: the fact that there is little label in noduli implies that the G10011 columnar neurons should not belong to the vertical system; to confirm: is there label in the gall/rubus (dorsal part of LAL)? Rx-positive cells described by authors in a previous publication do have projections to gall.

If the Tribolium embryonic progeny of DM1-DM4 is comparable to that of Drosophila DM1-DM4, the columnar neurons that we describe would be of the pontine type (PB-FB) Andrade et al., 2019. We do not discuss this as our analysis is not detailed enough for hard statements.

2.Ring neurons: is that term used in beetles? The corresponding neurons have been described for dung beetle, I believe. Given that the beetle EB is not ring-shaped, the axonal projection of "ring" neurons is also not circular. The problem of nomenclature should be discussed.
We feel that a discussion of the nomenclature is beyond the scope of our manuscript.

3.AOTU neurons: I am not clear on this group. Do the neurons have dendritic arbors in the AOTU, or are just cell bodies located close to it? Were neurons with the observed pattern described before, for beetles or Drosophila?
We think we see dendritic arbors in the AOTU. Please refer to the original image stacks that are accessible at figshare (see material and methods). We are not aware of any publication which describes cells with exactly the same position, morphology and elaborations.

Line 328ff: the paper Boyan et al. 2017 also describes comparatively embryonic FBpr development
added

Line 401: is the EB detectable with other markers in late larvae, like synapsin or acTub?
It is not detectable with Synapsin. We have not tried acTubulin. We provide this information now in the manuscript. Note that we have added arrows to Fig. 4H,H' to highlight the fibers which extend from the ring neurons and project towards the future EB.

Fig.6: I am basically convinced of the authors' statement of coincidence of skh and G10011. What would help in panel A is to outline (hatched line…) the brain hemispheres to demonstrate that skh, and G10011, are both expressed in a distinct small region of the overall brain.
We now provide additional data for the coincidence of skh and GFP RNA. These data are presented in Figure S5 C-C''.

What explains the different "textures" of the signals in panels B, B', B''? the skh RNA is more concentrated in the center of the cell group (these are all somata, right?), the GFP RNA in the periphery. Could the authors comment on that?
Well, we actually don't know the reason for the unusual distribution of the GFP RNA. We do a lot of in situs in our lab and most transcripts show a distribution similar to the skh RNA distribution. By contrast, GFP transcripts are routinely found at the periphery. Since GFP is normally not part of the Tribolium repertoire there should be no mechanism for sorting this RNA.

Reviewer 3 Advance summary and potential significance to field
The manuscript by Garcia-Perez et al describes the identification and functional characterization of the Shaking hands gene in Tribolium castaneum. This study is very carefully executed, nicely documented and reveals a role of the Shaking hands gene-encoded protein as a terminal neuronal differentiation factor in a subset of neurons that form the central complex. This study makes elegant use of the genetic tools available in Tribolium to start to decipher the genetic control of central complex development thereby also opening the possibility to start a systematic comparative analysis with Drosophila melanogaster where central complex development has been studied most extensively.

Reviewer 3 Comments for the author

© 2021. Published by The Company of Biologists under the terms of the Creative Commons Attribution License (https://creativecommons.org/licenses/by/4.0/).

1. On lines 74-75, the authors state that a role for terminal selectors has not been demonstrated yet in Drosophila. I do not think this is correct (e.g. the work of Stefan Thor and others). Please verify and adjust this statement as needed.
The reviewer is correct that terminal selectors have been studied in the ventral nerve cord by Thor and others. In our manuscript, we state that no role for terminal selectors has been demonstrated as yet in the Drosophila brain. We think this statement is correct. Nevertheless, we have removed this sentence.

2. On page 3-5, the authors give a very lengthy description of the central complex in Drosophila. I understand why this was done, given the extensive description of Drosophila CX anatomy. However, it would be very useful to also specify the (dis)similarities of the T. castaneum CX versus Drosophila. One example is the shape of the ellipsoid body.

We don't provide any detailed comparison of the CX anatomy Tcas versus Dm in this manuscript since nearly all our data describe the skh in Tcas. A future manuscript which will present more Dm data will also contain a detailed comparison of the CX anatomy. We feel that a more detailed comparison is beyond the scope of our current manuscript.

3. In line with the previous comment, the authors state on lines 112-113 that the architecture of the adult CX is well documented in many insect species. However, in a later statement they only refer to Drosophila and locust work (lines 247-251). Can the authors clarify this? When saying many insect species, how many? Different insect orders?

We now have added references which present studies in other insect species.

4. On line 188 (and elsewhere in the manuscript) the authors refer to n-dorsal or n-ventral brain. What does the n stand for?

"n" indicates directions according to the neuraxis, instead of the body axis

5. On lines 192-200, the authors argue that based on cell body location (and I assume size) that the neurons in the pars intercerebralis are neurosecretory and those in the tritocerebrum are likely to be hugin-expressing cells.
In the absence of positive labeling for neurosecretory cells and even more so for hugin, please provide more rationale. Is hugin encoded in the Tribolium genome? It would be more convincing to do a double-labeling experiment (e.g. immuno and in situ).

The reviewers are correct to state that we have not proven the neurosecretory character of these cells. However, these Tribolium neurons share more than their position, morphology and projections with the corresponding Drosophila cells: they have the same NB origin and express the same molecular markers. These data are not included in our manuscript as we feel it would take the focus of the CX. In our manuscript, we name these cells putative neurosecretory. We feel this statement is justified.

We have now checked whether Drosophila hugin has an orthologue in Tribolium: indeed, there is none. We have removed the term hugin from the manuscript and describe the respective cells as "large cells of the Tritocerebrum with ascending axon projections".

6. On line 255, the authors state: 'four characteristic fiber tracts commonly named ...'
Why commonly?

rephrased.

7. On line 274, the sentence is a bit strange. 'Embryonic are given oordinations according to the body axis'. I am not sure the word oordination exists. Is meant here, 'Embryonic stages are given coordinates according to the body axis'? Please clarify.

Yes, we do mean coordinates and have changed our wording accordingly.

8. In the legend of figure 3, there are a few typos and one term that I am not sure of.

© 2021. Published by The Company of Biologists under the terms of the Creative Commons Attribution License (https://creativecommons.org/licenses/by/4.0/).

The authors refer to 'stomodeum'. Is that the correct name at this developmental stage? Or should it be foregut?

At this developmental stage, the names "stomodeum" and "foregut" are used interchangeable. In all publications of the Bucher group, we consistently use the name "stomodeum".
Typos:
line 296: GFP-positive fascicles THAT enter the commissural ...
line 303: Multiple GFP-positive fibers exist IN the brain ...

These typos have been corrected.

9. In figure 3, please consider replacing the dark blue color in panel K by white or grey. I am not sure it will reproduce well. At least I was not able to distinguish the label very well.

We have changed the dark blue to bright magenta.

10. Line 312: small clusters OF GFP-positive ...

corrected

11. Lines 323-327: How do you come to the conclusion that G10011-GFP is expressed in the progeny of about 15% of all NB lineages?

This part has been removed.

12. Line 359: putative HUGIN expressing cells

Corrected (the term HUGIN has been removed as there is no HUGIN orthologue in Tribolium).

13. Line 441: FAITHFULLY

corrected

14. Line 445: It would be nice to include in Fig. S6 a protein sequence alignment, in particular of the Tribolium and Drosophila proteins.

We now show an alignment for the Tribolium and Drosophila proteins in Figure S6 B.

15. Lines 464-467: The antibody against Repo that was used to label glia. Since this is generated against the Drosophila Repo protein, how was this validated for use in Tribolium? Does it recognize a protein of the correct size in Tribolium (tested on Western blot)?

We have performed an extensive study addressing the cross-reactivity of Drosophila antibodies with Tribolium proteins. Our results (including the anti-Repo results) are published in Koniszewski et al., 2016. The specificity of Drosophila anti-Repo in labelling all Tribolium glia (except for midline-derived glia) was validated by combining Tc-repo-RNA in situ with rabbit anti-Repo immunostaining. We observed complete colocalization.

16. Lines 494-496. Do the axon outgrowth defects correlate with knockdown efficiency? Was this quantified?

We routinely observe a reduction of the mRNA abundance by 80-90% (when using a concentration of 2ug/ul dsRNA for injection as in the experiments presented in this manuscript). However, we have to concede that we did not perform qPCR for the skh knock-down.

17. Why was the analysis of Dm skh limited to the embryo? Would in situ analysis on larval (e.g. L3) and adult brain not provide further corroborating evidence? In particular given the link with the central complex, parts of which develop only later in development in Drosophila.

© 2021. Published by The Company of Biologists under the terms of the Creative Commons Attribution License (https://creativecommons.org/licenses/by/4.0/).

The reviewer is entirely correct. A thorough analysis of SKh expression and function in Drosophila at different developmental stages would corroborate and extend our Tribolium results. However, as it stands, our current manuscript is pushing the limits with respect to the length of the text and number of figures. The Drosophila data will be submitted for publication at a later time.

Second decision letter

MS ID#: DEVELOP/2020/199368

MS TITLE: The homeodomain transcription factor Shaking hands controls axon outgrowth of central complex neurons in the insect model Tribolium

AUTHORS: Natalia Carolina Garcia-Perez, Gregor Bucher, and Marita Buescher

I have now received all the referees reports on the above manuscript, and have reached a decision. The referees' comments are appended below, or you can access them online: please go to BenchPress and click on the 'Manuscripts with Decisions' queue in the Author Area.

The overall evaluation is positive and we would like to publish a revised manuscript in Development, provided that the referees' comments can be satisfactorily addressed. Please attend to all of the reviewers' comments in your revised manuscript and detail them in your point-by-point response. In particular, the reason that I am not accepting this revised MS outright is that it appears that you have not addressed all of the concerns of Reviewer 2 - the comments of that reviewer that appear unaddressed should be quite straightforward to deal with, and so I request that you please provide a response to each of these comments. If you do not agree with any of their criticisms or suggestions explain clearly why this is so.

We are aware that you may currently be unable to access the lab to undertake experimental revisions. If it would be helpful, we encourage you to contact us to discuss your revision in greater detail. Please send us a point-by-point response indicating where you are able to address concerns raised (either experimentally or by changes to the text) and where you will not be able to do so within the normal timeframe of a revision. We will then provide further guidance. Please also note that we are happy to extend revision timeframes as necessary.

Reviewer 1

Advance summary and potential significance to field

Please see my previous review.

Comments for the author

The authors did not fully address my major concerns:

The authors have only partially addressed my concerns regarding the co-expression of the G10011-GFP line and Tc-skh. The authors state that the G10011-GFP line “faithfully reports” skh expression based on co-expression studies in the embryo and adult. While the images for the embryonic co-expression are now convincing (two additional embryonic stages shown in Suppl. Fig. 5C of the revised ms, including the low expression in the stomodeum), the images of the adult brain (Fig. 6C) have not been changed and do not allow for evaluating if skh transcripts and GFP protein are co-expressed in all cells. The authors should present high resolution images that clearly show that skh transcripts and GFP protein are co-expressed in all cells or remove the sentence referring to the expression in the adult
‘All GFP-positive cells are also skh RNA positive, demonstrating the continued expression of skh (Fig. 6C-C’’)’.

© 2021. Published by The Company of Biologists under the terms of the Creative Commons Attribution License (https://creativecommons.org/licenses/by/4.0/).

One of my major concerns was about the analysis of the skh RNAi phenotype being superficial. Considering that the authors have put in a lot of work into characterizing the expression of the G10011-GFP line with regard to the formation of the central complex, I had expected a more detailed description of the RNAi phenotype. Specifically, I asked whether the progeny of DM1-DM4, which establish the primordium of the fan-shaped body, are present in the RNAi embryos, if so whether they extend axons and whether they fasciculate. The authors's answer was 'yes' to all questions. They also pointed out they could not observe if the axons defasciculate because "skh RNAi animals die at late embryogenesis and/or shortly after larval hatching". However, they have only added one sentence to the results
'Axon outgrowth defects were observed in all GFP-expressing neurons including the progeny of DM1-DM4, which in wild type generate the prFB.' There is no image supporting this statement. The authors should show images of the DM1-4 progeny their fasciculated axons and outgrowth defects and add the information (including the information that embryonic lethality prevents the analysis of later phenotypes) in the text of the results section.

A detailed response to my comment above would also cover an additional concern that has not been addressed. I stated in my previous review "Also with regard to RNAi: the authors state "Loss of Tc-Skh has drastic consequences for the outgrowth of all G10011-GFP positive axons: in severely affected embryos, no contra-laterally projecting axons enter the commissural system and hence, no prFB is formed". Since a (GFP negative) commissure is still seen in skh RNAi, the authors would have to show (1) that the progeny of DM1-4 do not extend axons and (2) that the FB is absent in the adult."
I also asked the authors about the DV levels shown in Fig. 8. "When presenting control and RNAi phenotypes, the authors should take care to show the same DV levels (e.g. Fig. 8A and C are clearly not showing the same DV level). "The authors' response (below) about the accuracy of the DV levels and the availability of the unprocessed images in figshare should be included in the figure legend.
"Panels 8A and C are both focused to show the fibers that enter the commissural system. Since no two brains are ever mounted in a perfectly equal manner a slight shift in the DV levels is common. To give all readers the opportunity to view the original unprocessed image stacks we have uploaded them to figshare."

Reviewer 2

Advance summary and potential significance to field

This manuscript reports the expression of a new homeobox gene, skh, in Tribolium and Drosophila. The authors provide a detailed, very lucid description of how expression relates to different elements of the central complex (CX), and they pay attention to different developmental stages from embryo to adult. The describe the expression of the Drosophila homolog in the embryo, and conduct knockdown studies in Tribolium which yield an interesting, interpretable phenotype. The paper makes a significant contribution to our understanding of brain development in Tribolium, and insects in general.

Comments for the author

The authors have addressed the comments and suggestions of me and the other two reviewers in detail. I find the manuscript is acceptable for publication.

Reviewer 3

Advance summary and potential significance to field

The manuscript by Garcia-Perez et al describes the identification and functional characterization of the Shaking hands gene in Tribolium castaneum. This study is very carefully executed, nicely documented and reveals a role of the Shaking hands gene-encoded protein as a terminal neuronal differentiation factor in a subset of neurons that form the central complex. This study makes

© 2021. Published by The Company of Biologists under the terms of the Creative Commons Attribution License (https://creativecommons.org/licenses/by/4.0/).

elegant use of the genetic tools available in Tribolium to start to decipher the genetic control of central complex development thereby also opening the possibility to start a systematic comparative analysis with Drosophila melanogaster where central complex development has been studied most extensively.

Comments for the author

The authors have addressed a number of my concerns and provided rationale for why they did not include certain results.

Second revision

Author response to reviewers' comments

Response to reviewer 1:

We now provide additional images of an adult brain stained with skh RNA and anti GFP. The additional images have been added to Figure S5 and include panels with high magnification. Also, we have rephrased our statement that 10011 faithfully reportes skh RNA expression: (lines 295 to 297) of the main text. Most, and possibly all, *skh RNA* positive cells are also GFP-positive, demonstrating the continued expression *of skh* transcript. However, due to technical limitations, we cannot exclude that a small number of *skh RNA* positive cells do not express GFP.

We now provide additional images which clearly show that even in knock down embryos which have no GFP positive commissural fibers, GFP positive progeny of the neuroblasts DM1 DM4 is still present. Moreover, we show one example of the stalling of a fascicle produced by the DM4 progeny. These images have been added to Figure 8 (G-G''). (lines 320 to 322). Axon outgrowth defects were observed in all GFP-expressing neurons including the progeny of DM1-DM4, which in wild type generate the prFB (Fig. 8. G-G'').

In addition, we have rephrased our statement (lines 328 to 332): Loss of GFP expression in DM1-DM4 progeny occurs with low penetrance and hence, is unlikely to be the main cause for the loss of GFP-positive commissural fibers which constitute the prCX (of nine embryos with no GFP-positive commissural fibers only three showed loss of one or more GFP- positive DM1-DM4 clusters, Fig. 8G and data not shown).

We agree with the reviewer that the link to figshare should be provided in the legend to figure 8 (line 849) as well as under the heading **data availability**.

Third decision letter

MS ID#: DEVELOP/2020/199368

MS TITLE: The homeodomain transcription factor Shaking hands controls axon outgrowth of central complex neurons in the insect model Tribolium

AUTHORS: Natalia Carolina Garcia-Perez, Gregor Bucher, and Marita Buescher
ARTICLE TYPE: Research Article

I am happy to tell you that your manuscript has been accepted for publication in Development, pending our standard ethics checks.

© 2021. Published by The Company of Biologists under the terms of the Creative Commons Attribution License (https://creativecommons.org/licenses/by/4.0/).

Reviewer 1

Advance summary and potential significance to field

See previous reviews

Comments for the author

I am happy with the revision.

© 2021. Published by The Company of Biologists under the terms of the Creative Commons Attribution License (https://creativecommons.org/licenses/by/4.0/).
